# Dual-faced metabolic reprogramming powers proliferation: FGF21 drives lung squamous carcinoma progression via CaMKII-DRP1-mediated mitochondrial fission

**DOI:** 10.1038/s41419-026-09084-3

**Published:** 2026-07-16

**Authors:** Xiao-xin Pu, Yan Zhang, Bao-yi Liu, Xiao-jie Song, Yu-wen Xue

**Affiliations:** 1 Department of Pulmonary and Critical Care Medicine, Qilu Hospital (Qingdao), Cheeloo College of Medicine, Shandong University, Qingdao, China; 2 Department of Pulmonary and Critical Care Medicine, The fourth Hospital of Jinan, Jinan, China; 3 Department of Pulmonary and Critical Care Medicine, Qilu Hospital, Cheeloo College of Medicine, Shandong University, Jinan, China

**Keywords:** Non-small-cell lung cancer, Cancer metabolism

## Abstract

Fibroblast growth factor 21 (FGF21) is upregulated in lung squamous cell carcinoma (LUSC) tissues, cell lines, and tumor-conditioned media. Functional studies using LUSC cell lines (H1703, H520) demonstrate that FGF21 promotes cancer cell proliferation, migration, invasion, and tumor sphere formation in vitro. This pro-tumorigenic effect was validated in vivo through xenograft models, where intra-tumoral FGF21 administration accelerated tumor growth. Mechanistically, FGF21 activates calcium/calmodulin-dependent protein kinase II (CaMKII), which phosphorylates dynamin-related protein 1 (DRP1, encoded by DNM1L) at Ser616. Phosphorylated DRP1 translocates to mitochondria, inducing mitochondrial fission. Metabolic analyses revealed that FGF21 remodels cellular energetics in a cell type-specific manner, enhancing glycolysis in H520 cells and oxidative phosphorylation (OXPHOS) in H1703 cells, consistent with their inherent predominant metabolic states. Knockdown of DRP1 or pharmacological inhibition of CaMKII (KN93) abolished FGF21-driven mitochondrial fission, metabolic reprogramming, and tumor-promoting effects. Collectively, FGF21 acts as a tumor-promoting factor in LUSC by activating the CaMKII/DRP1-Ser616 axis to induce pathological mitochondrial fission and metabolic reprogramming, identifying this pathway as a potential therapeutic target.

## Highlights

1. FGF21 functions as a tumor-promoting factor in lung squamous carcinoma (LUSC), driving cancer cell proliferation and tumor growth in vitro and in vivo via its secretion.
2. Induction of mitochondrial fission and subsequent metabolic reprogramming by FGF21 fuels LUSC advancement, and the effects vary across different cell types.
3. FGF21 triggers mitochondrial fission and metabolic reprogramming primarily through activation of the CaMKII-DRP1 signaling axis.

## Introduction

Lung cancer remains the leading cause of the cancer mortality worldwide, accounting for ~20% of cancer-related deaths [1]. Non-small cell lung cancer (NSCLC), accounting for 85% of all lung cancer cases [2], encompasses distinct histological subtypes, with lung squamous cell carcinoma (LUSC) accounting for 20–30% of the lung cancer diagnoses. Despite declining smoking rates in developed nations [3, 4] and improved treatments, LUSC has a poor 5-year survival rate. Thus, novel therapies are urgently needed [1, 2].

The fibroblast growth factor (FGF) family comprises 23 different FGF ligands. FGF signaling has been shown to be dysregulated in many diseases, including different lung diseases. Evidence supports the protective activity of FGFs in lung diseases, including idiopathic pulmonary fibrosis (IPF) [5, 6], pulmonary arterial hypertension [7], ventilator-induced lung injury [8] and lung cancers [9]. Interestingly, their functions diverge in carcinomas, for example, FGF2 and FGF19 consistently promote tumorigenesis across multiple cancers (e.g. breast, gastric, osteosarcoma, and colorectal carcinomas) [10–14], while FGF21 exhibits both pro-and anti-tumorigenic activities in carcinomas [15–19]. FGF21 signals primarily through FGFR1, a receptor amplified in 20% of LUSC cases [20, 21], providing a prevalent genetic basis for FGF21 activity in LUSC. However, the role and mechanisms of FGF21 in LUSC remain poorly understood.

Increasing evidence highlights the pivotal role of FGF21 in metabolic regulation [22–24], with its mitochondrial interactions increasingly studied across diverse diseases [25–29]. Given that metabolic reprogramming is a hallmark of cancer progression, this process extends beyond the well-known Warburg effect (aerobic glycolysis), with recent studies confirming that mitochondrial OXPHOS is a critical metabolic process for tumor growth [30]. As the central hub for OXPHOS, mitochondria dynamically remodel their morphology through fission/fusion events to meet energy demands [31]. This structural plasticity, governed by fission executors such as DRP1 and fusion mediators (MFN1/2, OPA1) [32], directly impacts functional adaptability. Although mitochondrial dynamics are strongly associated with cancer pathogenesis [33–36], their role in LUSC remains underexplored. Building on the broad relevance of FGF21-mitochondrion crosstalk, our bioinformatics analysis revealed that elevated FGF21 levels in LUSC tissues are correlated with mitochondrial-associated gene signatures. We therefore hypothesized that FGF21 promotes LUSC growth by modulating mitochondrial dynamics to reprogram cellular metabolism.

Here, by using bioinformatics, cell line models, clinical samples, and xenograft systems, we comprehensively examined FGF21 expression patterns in LUSC. We further evaluated its functional impact on tumor progression and mitochondrial dynamics. More importantly, the mechanistic cascade linking FGF21 signaling to DRP1-mediated mitochondrial fission and the resulting consequences for metabolic reprogramming were explored.

## Results

### FGF 21 is upregulated in LUSC tissues, cell lines and tumor-conditioned medium (TCM)

**Fig. 1:**
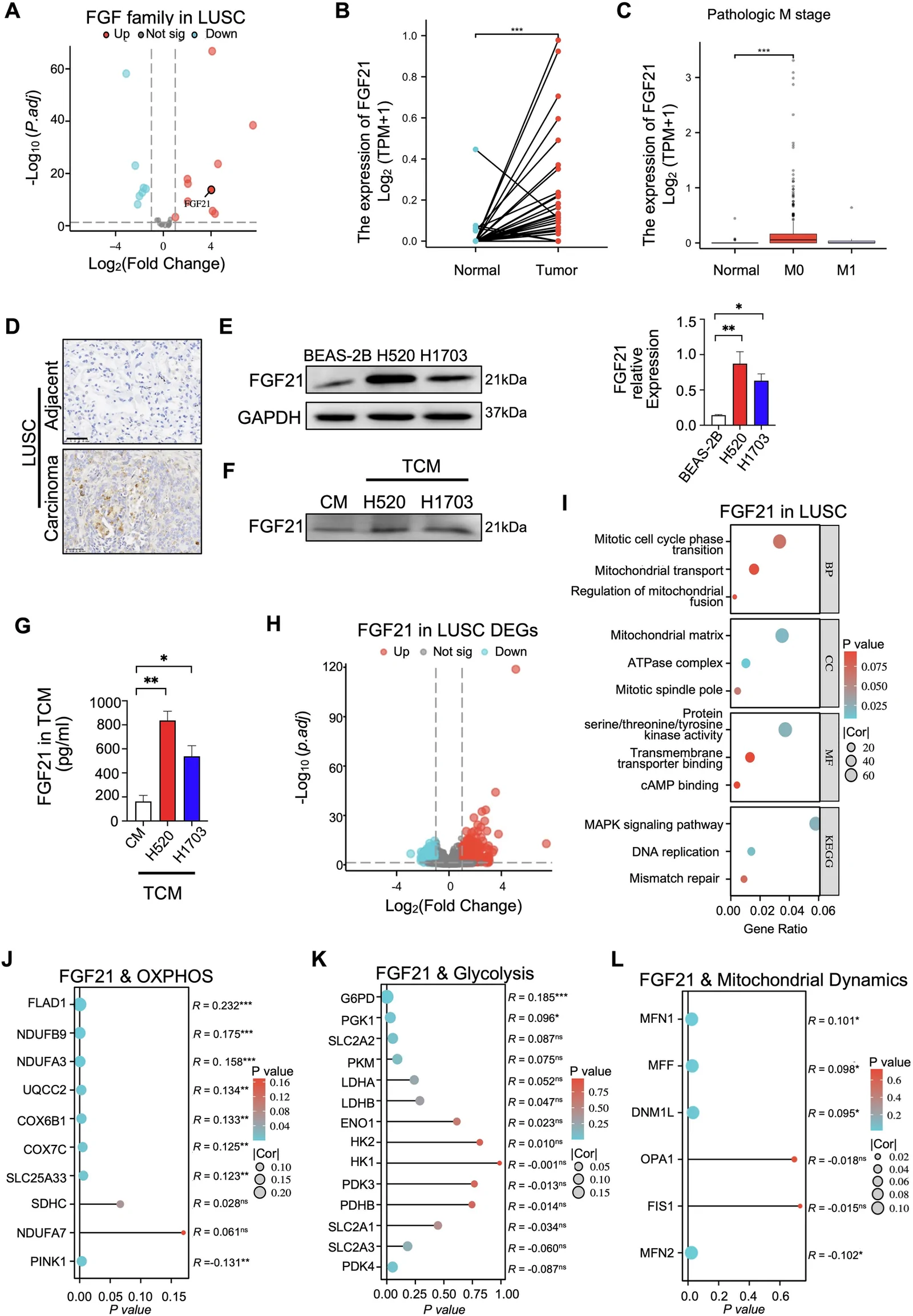
FGF21 is upregulated in LUSC tissues, cell lines and TCM. **A**–**C** The expression of FGF family members and FGF21 in LUSC and adjacent tissues was analyzed using TCGA data. **D** Representative microphotographs of IHC staining of FGF21 (brown) in LUSC tissues versus adjacent tissues; scale bars, 50 μm. **E**, **F** Whole-cell lysates (**E**) and concentrated TCM/CM (**F**) from the indicated LUSC cell lines and normal human bronchial epithelial cells (BEAS-2B) were analyzed by western blotting. For whole-cell lysates, equal amounts of total protein determined by BCA assay, were loaded per lane. For TCM/CM, conditioned medium was collected, concentrated by methanol-chloroform precipitation, and normalized by total protein concentration (BCA assay) prior to loading to ensure equal loading, as classical intracellular loading controls (e.g., GAPDH, β-actin) are absent in cell-free supernatants. **G** FGF21 levels in TCM/CM were quantified via ELISA. **H** Volcano plot of DEGs associated with FGF21 in LUSC tumor samples. **I** GO and KEGG pathway analyses of FGF21-related genes in LUSC. **J**–**L** Lollipop plots depicting the expression of oxidative phosphorylation-, glycolysis-, and mitochondrial dynamics-related genes linked to FGF21 in LUSC. The data are presented as the means ± standard errors of the means (SEMs) or standard deviations (SDs); *p < 0.05, **p < 0.01, ***p < 0.001.

To assess the expression of ligand family-related FGFs, we first investigated the expression of members of the FGF family in LUSC samples using the Cancer Genome Atlas (TCGA) database (https://portal.gdc.cancer.gov/). RNA-seq analysis revealed that the mRNA levels of FGF21 were significantly greater in LUSC tissues than in normal tissues (Fig. 1A, B), which was corroborated by a pancancer analysis (Fig. S1A).

Furthermore, our stage-stratified analysis revealed that FGF21 upregulation occurs exclusively in primary M0-stage LUSC (nonmetastatic tumors) (Fig. 1C). Bioinformatic analyses revealed divergent associations between FGF21 expression and clinical outcomes in LUSC. In the TCGA primary dataset analysis, elevated FGF21 expression showed a borderline significant association with prolonged overall survival (OS; log-rank *p* = 0.04) but not the progression-free interval (PFI; log-rank *p* = 0.054). However, independent validation through the UALCAN platform revealed no significant correlation between FGF21 expression and OS (log-rank *p* = 0.05). These results indicate that the prognostic value of FGF21 expression is dataset dependent (Fig. S1B–D). Our findings show that abnormal FGF21 levels arise early in tumor development—specifically, when cancer cells first establish themselves locally before gaining the ability to spread to lymph nodes or distant organs.

We also aimed to validate these data by assessing the expression of FGF21 in LUSC using clinical samples from patients admitted to our hospital. However, the serum FGF21 concentration was not significantly elevated in LUSC patients (*n* = 30) compared with healthy donors (*n* = 10; Fig. S1E, Table S1), while the tissue-level analyses revealed distinct patterns. In the clinical samples from our patient cohort, immunohistochemistry (IHC) revealed strong FGF21 staining primarily within the tumor matrix of LUSC tissues (Fig. 1D). Given the known heterogeneity of LUSC, we further profiled FGF21 expression across cell lines derived from both primary (H520) and metastatic (H1703) origins. Western blot analysis confirmed elevated FGF21 expression in LUSC cell lines compared with that in normal bronchial epithelial cells (BEAS-2B; Fig. 1E), and critically, treatment with concentrated TCM resulted in significantly greater FGF21 secretion than did treatment with control media, as determined via both immunoblotting after protein concentration quantification, as described by Jacobs [37] (Fig. 1F), and enzyme-linked immunosorbent assay (ELISA) quantification (Fig. 1G). This consistent elevation in cellular and secretory FGF21 levels suggests active autocrine signaling within the tumor microenvironment, which contrasts with systemic circulation patterns.

To further explore the role of FGF21 in the malignant properties of LUSC, we performed differentially expressed gene (DEG) analyses. Using a fold change of ≥1.0 and *P* < 0.05 as the criteria, 878 DEGs were identified, and among the DEGs, 690 were upregulated and 188 were downregulated in LUSC patients with upregulated FGF21 expression in a TCGA dataset (Fig. 1H, Table S1). Interestingly, the Gene Ontology (GO) and Kyoto Encyclopedia of Genes and Genomes (KEGG) analyses indicated that the DEGs were involved in mitochondrial functions in LUSC (Fig. 1I). To further clarify whether FGF21 influences mitochondrial function in LUSC patients, we analyzed the correlations between the expression of mitochondrial OXPHOS-, glycolysis- and mitochondrial dynamics-related genes and FGF21. The data were visualized (Fig. 1J–L). Moreover, we investigated other genes related to mitochondrial function (Fig. S1F). The data showed that the expression of FGF21 is related to mitochondrial function. These results suggest that FGF21 upregulation in LUSC may be linked to mitochondrial dynamics and metabolic reprogramming.

Collectively, FGF21 is upregulated in LUSC tissues, cell lines and TCM. Based on this and bioinformation results, proposes that FGF21 may influence tumor malignancy in LUSC and potentially alter its metabolic patterns through mitochondrial mechanisms.

### In vitro FGF21 promotes the proliferation and metastatic capacity of LUSC cells

**Fig. 2:**
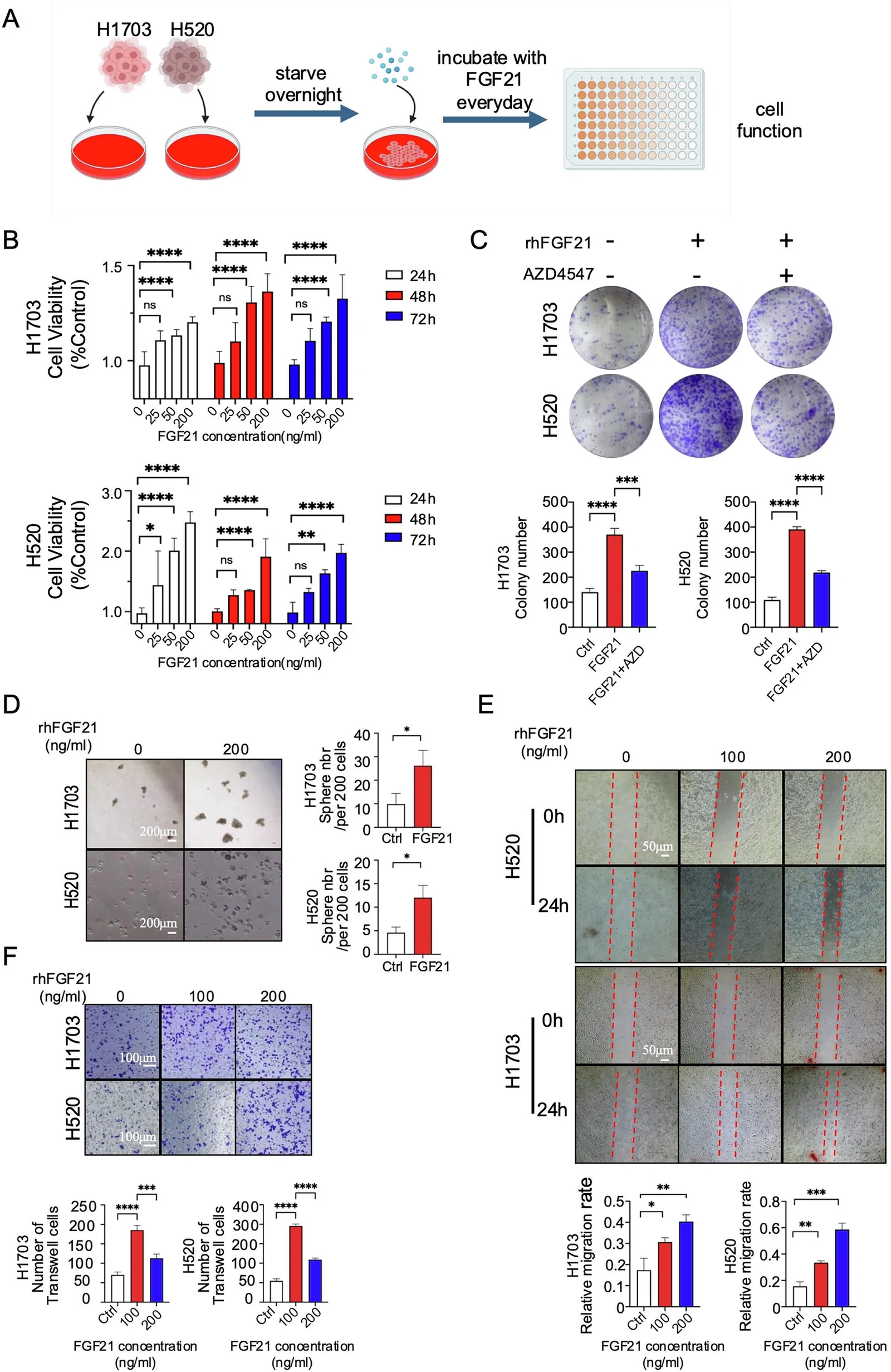
In vitro FGF21 promotes malignant phenotypes of LUSC cells. **A** Schematic workflow of the experimental design. **B** Dose- and time-dependent effects of FGF21 (25–200 ng/mL) on the viability of H1703 and H520 cells at 24 h, 48 h, and 72 h. **C** Colony formation capacity of cells treated with FGF21 and/or AZD4547. **D** Tumor sphere formation in serum-free medium supplemented with/without FGF21; scale bars, 200 μm. **E** Wound healing assay showing the lateral migration rates; scale bars, 50 μm. **F** Transwell invasion assay demonstrating the vertical migration rates; scale bars, 100 μm. The data are presented as the means ± SEMs (*n* = 3). *p < 0.05, **p < 0.01, ***p < 0.01, ****p < 0.0001.

To validate bioinformatic predictions regarding the tumor related potential of FGF21, we conducted functional characterization in two histologically distinct LUSC cell lines—metastasis-derived H1703 cells and primary tumor-derived H520 cells—to represent LUSC heterogeneity (Fig. 2A). We examined the role of FGF21 in basic cell behaviors in vitro, including cell proliferation, invasion, and migration.

Quantitative assessment of cellular proliferation via CCK-8 assays revealed a dose- and time-dependent increase in proliferation in response to FGF21 stimulation (25–200 ng/mL) (Fig. 2B). Notably, even at a dose of 50 ng/ml, sustained treatment for 24 h elicited significant increases in the number of viable cells in both cell lines, suggesting persistent activation of proliferative signaling cascades. Mechanistically, FGF21 activated FGFR1 phosphorylation, whereas the FGFR inhibitor AZD4547 attenuated this activation in both lines (Fig. S2A). This signaling axis critically affects functional outcomes, as chronic FGF21 exposure (200 ng/mL, 21 days) dramatically increased colony formation efficiency—an effect completely abolished by AZD4547 cotreatment (Fig. 2C). Interestingly, FGF21 markedly potentiated tumor sphere formation under serum-low suspension culture conditions, with treated cells exhibiting both quantitative increases in sphere count and diameter, implying functional amplification of cancer stem cell-like properties (Fig. 2D). Wound healing assays revealed accelerated lateral migration at 24 h posttreatment (Fig. 2E). Transwell assays confirmed enhanced early-stage Matrigel invasion (24 h) (Fig. 2F).

Given the contrasting observations between the pro-proliferative role of FGF21 in vitro and its association with OS revealed via bioinformatic analysis, we also analyzed the effects of FGF21 on immune infiltration across different types of cancer (Fig. S3A), including LUSC (Fig. S3B–D). The proportions of immune infiltrating cells and the expression of immune checkpoint-associated genes were analyzed. The results revealed significant associations between FGF21 expression and the proportions of Tγδ cells and Th2 cells within the LUSC microenvironment.

In summary, the results derived from in vitro experiments suggest that FGF21 plays a protumor role in LUSC cells.

### Mitochondrial fission and metabolic reprogramming is activated by FGF21 in LUSC cells

**Fig. 3:**
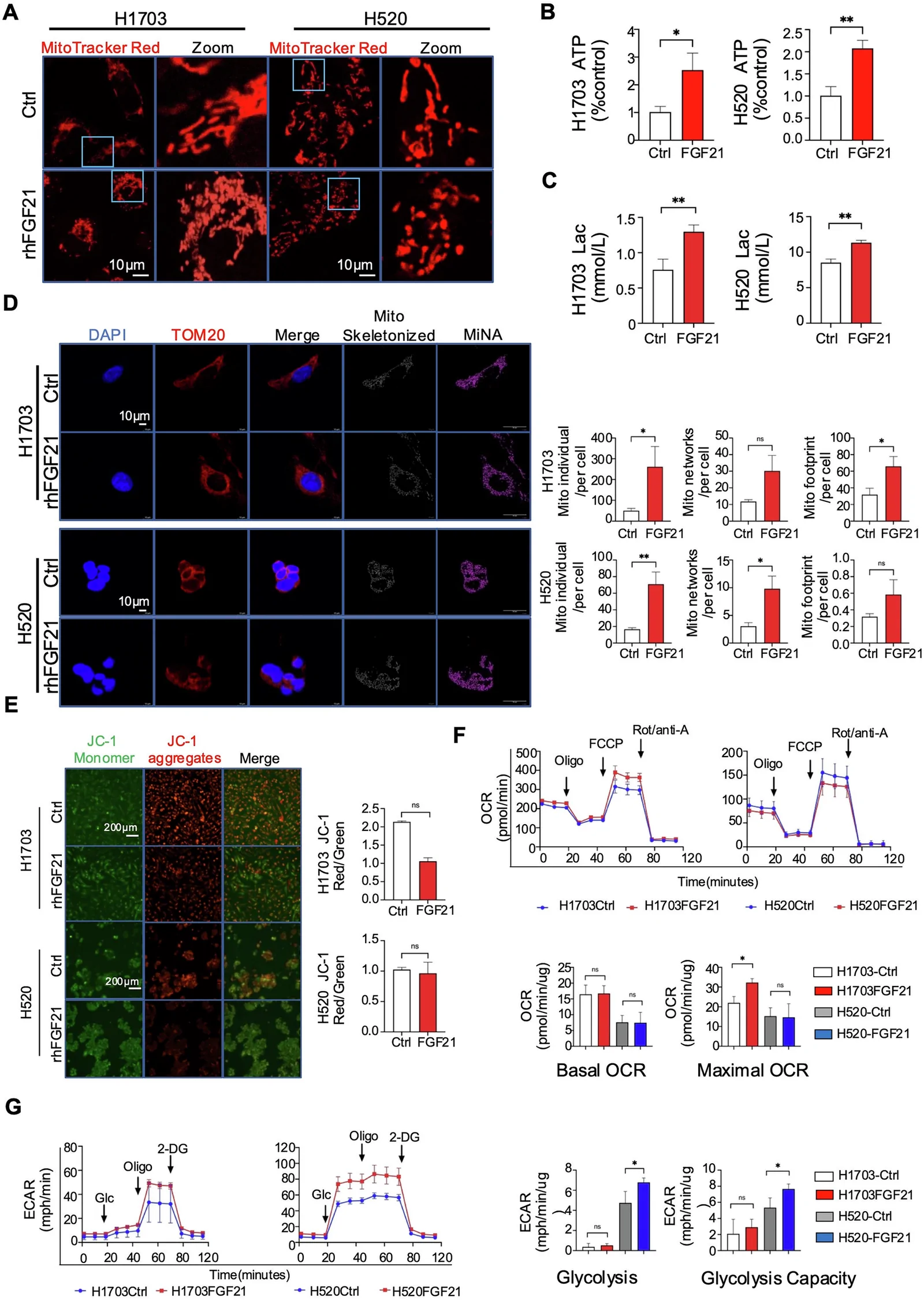
FGF21 promotes mitochondrial fission and metabolic reprogramming in LUSC cells. **A** Live-cell imaging of mitochondria using Mito Tracker Red in H1703 and H520 cells after 12 h of treatment with rhFGF21 (200 ng/ml); scale bars, 10 μm. **B** Total ATP levels in LUSC cells treated with or without rhFGF21 were measured via a luminescence assay. **C** Quantitative analysis of lactate levels in conditioned media from LUSC cells treated with rhFGF21. **D** TOM-20-mediated mitochondrial morphology analysis. The number of individual mitochondria, network and footprints per cell were quantified, *n* = 6, scale bars, 10 μm. **E** The MMP was quantified by JC-1 staining (monomers: green; aggregates: red) in H1703 and H520 cells pretreated with rhFGF21 using confocal microscopy; scale bar, 200 μm. **F**, **G** Metabolic flux profiling. The OCR and ECAR were assessed using a Seahorse XF analyzer. Sequential additions: Oligo (1 μM), FCCP (0.5 μM), and Rot/AA (rotenone/antimycin) (0.5 μM each) for OCR; Glc (glucose) (10 mM), Oligo (oligomycin) (1 μM), and 2-DG (100 mM) for ECAR. The data are shown as the means ± SEMs or SDs; *p < 0.05, **p < 0.01, ***p < 0.01.

On the basis of the bioinformatics results, we detected the influence of FGF21 on mitochondrial dynamics in LUSCs. First, we used Mito Tracker to label mitochondria in living cells and detected an increase in the number of mitochondria following incubation with rhFGF21 (200 ng/ml) relative to that in the controls (Fig. 3A). Notably, FGF21 stimulation increased both ATP generation (Fig. 3B) and lactate secretion (Fig. 3C), indicative of a hybrid metabolic state combining oxidative and glycolytic phenotypes. To eliminate the bias caused by the mitochondrial membrane potential (MMP) and correctly detect mitochondria, we also performed an immunofluorescence assay with TOM20, a recognized mitochondrial marker. Indeed, compared with that in the control groups, TOM20 staining in LUSC cells resulted in a similar increase in the number of mitochondria, which was in line with the Mito Tracker analysis results. The increase in the number of mitochondria in LUSC cells incubated with FGF21 was confirmed by quantitative analysis (Fig. 3D), while analysis of mitochondrial networks and footprint were differ in different cell lines. This pattern contrasts with classical mitophagy-associated fragmentation, suggesting alternative mitochondrial remodeling. To test whether this structural reorganization impaired function, we measured the MMP (ΔΨm) in LUSC cell lines via JC-1 staining. While FGF21-exposed cells presented subtle ΔΨm attenuation, this change was not statistically significant (Fig. 3E). Collectively, the preserved network integrity with increased organelle count, coupled with maintained ΔΨm, indicates that FGF21 induces highly efficient mitochondrial fission without compromising functional integrity.

To further assess the effect of FGF21 on metabolism in LUSC cells, we performed extracellular flux analysis to assess the oxygen consumption rate (OCR, indicating mitochondrial OXPHOS activity) and the extracellular acidification rate (ECAR, reflecting glycolytic lactate production). The results revealed that FGF21 enhances OXPHOS in LUSC cells, evidenced by increased OCR following FCCP treatment (Fig. 3F). Notably, the significant difference analysis showed that while H1703 cells exhibited higher basal OCR than H520 cells, FGF21 specifically elevated maximal respiratory capacity in H1703 without altering basal respiration. Concurrently, FGF21-treated cells showed heightened ECAR under oligomycin-induced glycolytic stress, with this effect being particularly pronounced in H520 cells, which displayed stronger basal glycolytic capacity compared to H1703 (Fig. 3G). These results establish that FGF21 drives metabolic reprogramming by boosting glycolysis and mitochondrial respiration depending on cell type-specific adaptations observed between H1703 and H520 lines.

Taken together, our data show that FGF21 drives LUSC proliferation by altering mitochondrial dynamics. This remodeling increases energy production efficiency to meet higher demands. Importantly, this process depends on the unique metabolic baseline of the cell type.

Notably, baseline metabolic profiling revealed that H1703 cells exhibit a significantly higher OCR/ECAR ratio compared with H520 cells, indicative of a greater reliance on oxidative phosphorylation (Fig. S6A). Consistent with this, analysis of Cancer Cell Line Encyclopedia (CCLE) data showed elevated expression of glycolytic genes (LDHA, PKM2, HK2, PFKM) in H520 cells (Fig. S6B), which may contribute to the cell-type-specific metabolic responses to FGF21.

### FGF21 enables mitochondrial translocation of DRP1 to drive mitochondrial fission in LUSC cells

Our studies revealed an increased number of mitochondria after treatment with FGF21 in LUSC cells. However, this event can be the outcome of two different processes: decreased mitochondrial fusion or increased mitochondrial fission. Therefore, we next investigated the underlying mechanisms in LUSC cells.

**Fig. 4:**
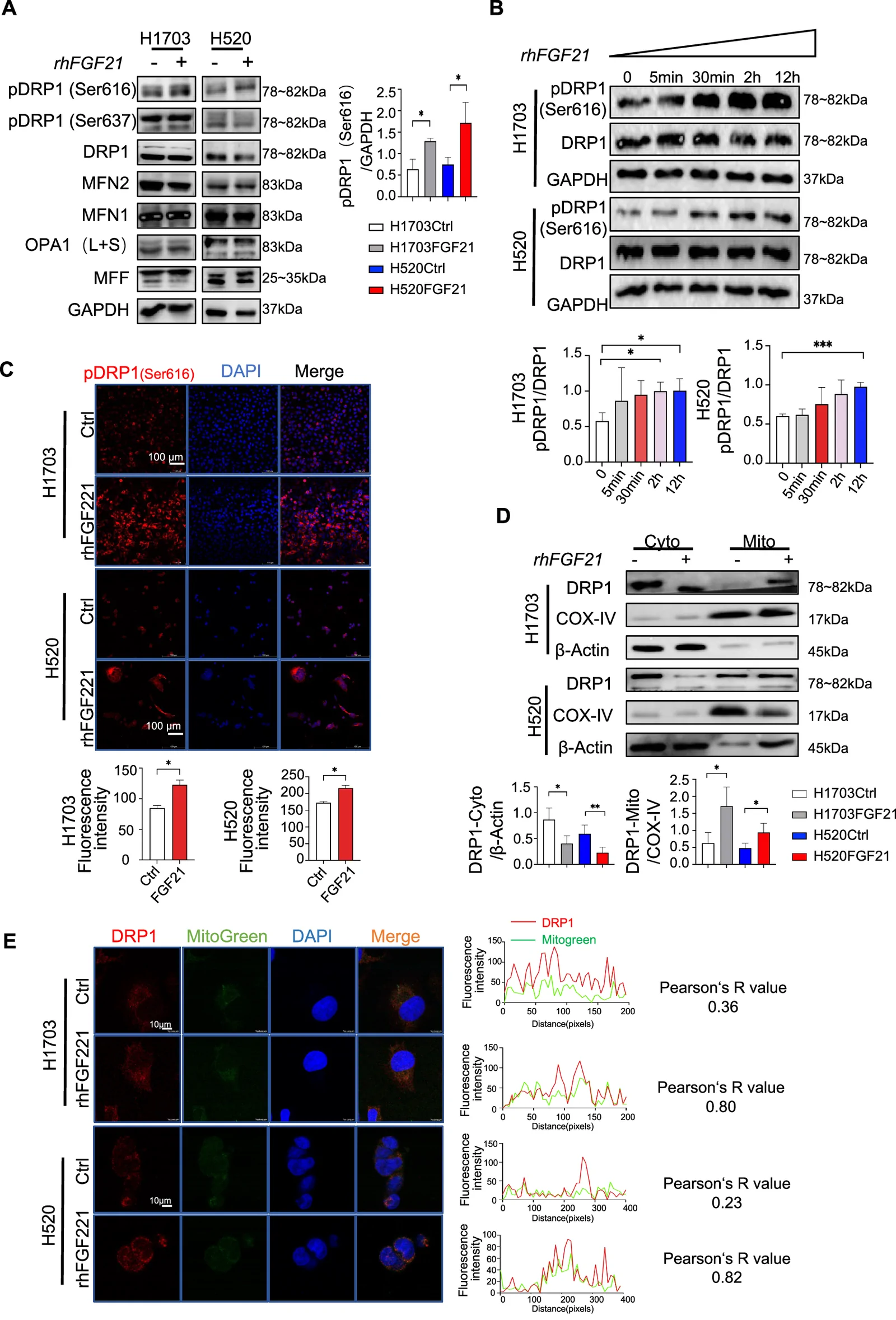
Mitochondrial translation of DRP1 mediates FGF21-induced mitochondrial fission in LUSC cells. **A** Representative Western blotting images showing the expression of mitochondrial fusion and fission proteins in response to FGF21 treatment. The protein levels were normalized to the GAPDH levels. **B** Representative Western blotting images showing the expression of p-DRP1 Ser616 and DRP1. **C** Immunofluorescence staining showing the expression level of p-DRP1 Ser616; scale bars, 100 μm. Fiji software was used for image analysis, and the average fluorescence intensity was calculated. **D** Fractions of LUSC cell lysates, showing the levels of DRP1 in the mitochondrial (Mito) and cytoplasmic (Cyto) fractions. The mitochondrial protein COX-IV and the cytoplasmic protein β-actin served as controls. **E** Confocal microscopy images showing the levels of mitochondria-bound DRP1 in LUSC cells treated with FGF21. DRP1 and mitochondria were labeled with a mouse anti-DRP1 antibody and Mito Tracker Green, respectively; scale bars, 10 μm (annotation added; original software-generated scale bar displayed a “too long” error due to a display glitch). Pearson’s coefficient indicates colocalization of DRP1 and Mito Tracker Green. The data are shown as the means ± SEMs or SDs; *p < 0.05, **p < 0.01, ***p < 0.01.

To evaluate mitochondrial dynamics in FGF21-treated LUSC cells, we systematically evaluated key regulatory proteins: fusion mediators (MFN1/2, OPA1) and fission executors (DRP1, MFF). Initial western blotting revealed almost no changes in fusion mediators and minimal changes in total DRP1/MFF levels in whole-cell lysates (Fig. 4A). Prompted by established links between DRP1-Ser616 phosphorylation and tumor progression [38], we examined site-specific DRP1 activation. Consistently, FGF21 stimulation in H1703/H520 cells selectively increased mitochondrial DRP1-Ser616 phosphorylation, as shown by Western blotting, further confirming the time-dependent increase in DRP1-Ser616 activation (Fig. 4B). In contrast, the DRP1-Ser637 levels remained unaltered, as shown in Fig. 4A. This phosphorylation pattern was visually confirmed through quantitative immunofluorescence (Fig. 4C). Subcellular fractionation definitively revealed FGF21-driven DRP1 enrichment in mitochondrial fractions with concurrent cytoplasmic depletion (Fig. 4D), whereas costaining revealed increased DRP1–mitochondria colocalization (Fig. 4E).

We also examined the expression of DRP1 in LUSC tissues (Fig. S4A, B, Table S3), and we conducted immunofluorescence colocalization with TOM20 and DRP1 (Fig. S4C). These findings suggest a close correlation between DRP1 mitochondrial translocation and tumor progression. Immunofluorescence colocalization (TOM20/DRP1) in clinical specimens revealed increased mitochondrial DRP1 accumulation in LUSC tissues compared with adjacent normal tissues (Fig. S4A–C), suggesting tumor-associated translocation.

Complementary time-course assays revealed no mitochondrial fission protein 1 (Fis1) upregulation under FGF21 treatment (Fig. S4D), indicating that FGF21-induced fission operates independently of Fis1-mediated peripheral pathways.

These results collectively indicate that FGF21 promotes DRP1 mitochondrial translocation via phosphorylation at Ser616.

### DRP1 plays a pivotal role in FGF21-driven proliferation, mitochondrial fission, and metabolic reprogramming in LUSC

**Fig. 5:**
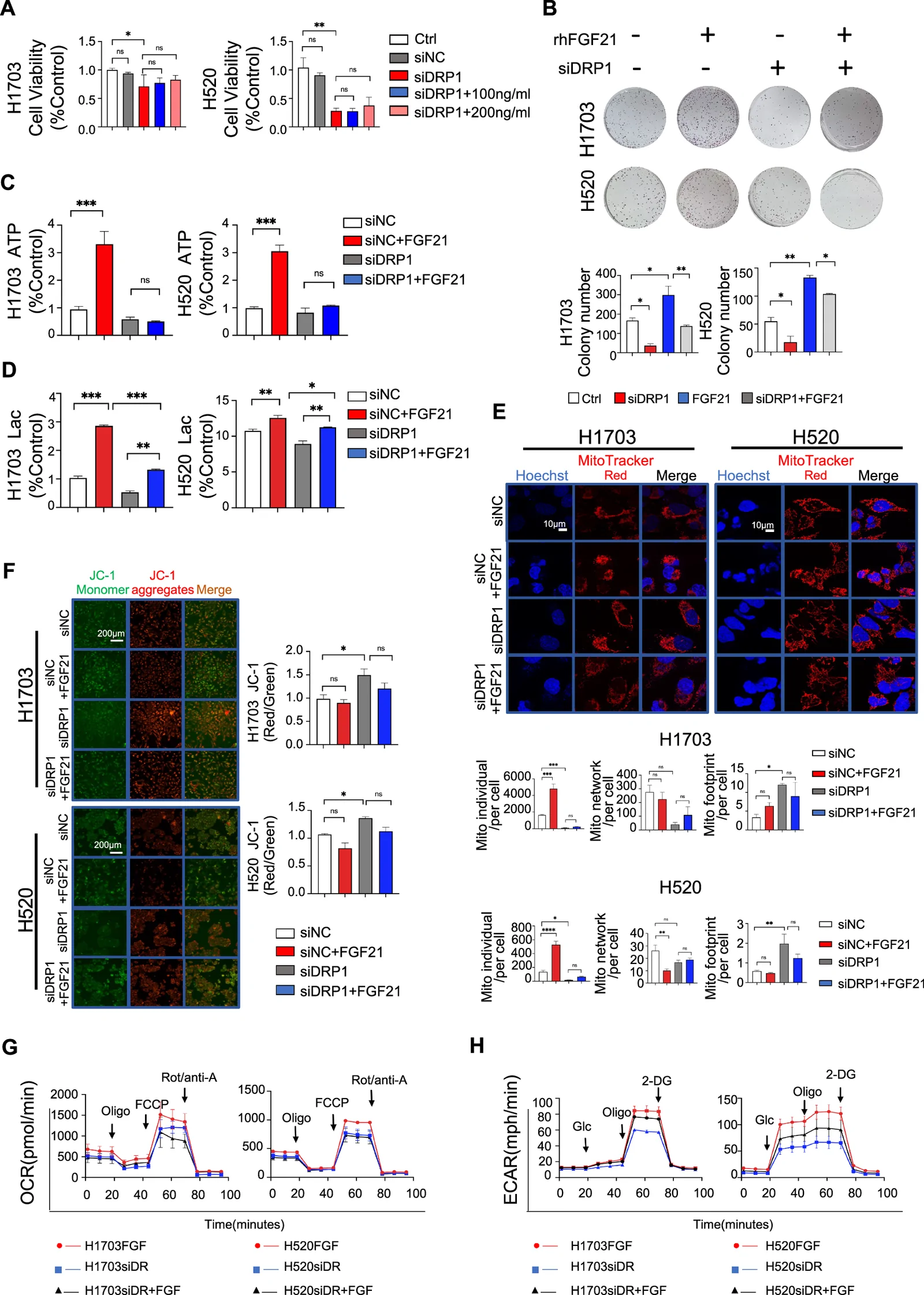
Suppression of DRP1 reverses the pro-tumorigenic effects of FGF21 on proliferation and metabolism in LUSC. **A** CCK-8 assay showing the viability of DRP1-silenced H1703 and H520 cells with or without FGF21 treatment. **B** Colony formation capacity and viability of DRP1-silenced H1703 and H520 cells with or without FGF21 treatment. **C**, **D** Intracellular ATP levels and lactate production in DRP1-silenced cells treated with rhFGF21 (200 ng/ml, 24 h). **E** Immunofluorescence staining of mitochondria (Mito-Tracker Red) and nuclei (Hoechst33342) in H1703 and H520 cells treated with rhFGF21 (200 ng/ml, 12 h) after DRP1 siRNA transfection; scale bars, 10 μm. The individual mitochondria, network and footprints per cell were quantified. **F** MMP was assessed by JC-1 staining in DRP1-silenced H1703 and H520 cells treated with rhFGF21; scale bars, 200 μm. **G**, **H** Metabolic flux rescue. **G** The OCR and **H** ECAR profiles of DRP1-silenced LUSC cells treated with or without FGF21 were analyzed using the Seahorse XF platform. The data are shown as the means ± SEMs or SDs; *p < 0.05, **p < 0.01, ***p < 0.01, ****p < 0.01.

To define the functional contribution of DRP1-mediated mitochondrial fission to LUSC pathogenesis, we established a DRP1-knockdown model via siRNA transfection, and effective protein depletion was confirmed by immunoblotting (Fig. S4A). Having validated the model, we assessed the role of DRP1 in FGF21-driven oncogenesis through proliferation assays. CCK-8 and colony formation analyses consistently demonstrated that DRP1 silencing attenuated the pro-proliferative effects of FGF21 in LUSC cells (Fig. 5A, B).

To further examine how DRP1 regulates FGF21-induced metabolic alterations, we quantified cellular bioenergetic outputs. Notably, while FGF21 typically increases ATP production, DRP1 knockdown suppressed cellular ATP levels despite FGF21 stimulation (Fig. 5C). Compared with FGF21 treatment alone, DRP1 silencing partially reversed FGF21-driven lactate overproduction (Fig. 5D). These findings establish the significant role of DRP1 in the metabolic shift. Notably, lactate levels remained elevated in DRP1-silenced cells treated with FGF21 compared with DRP1-silenced cells without FGF21 treatment. This persistent increase was statistically significant. These findings suggest that complementary pathways mediate the regulation of lactate by FGF21. Subsequent ECAR measurements confirmed these findings.

Mechanistically, DRP1 deficiency profoundly altered mitochondrial architecture. As visualized by morphological analysis, DRP1-silenced cells presented elongated mitochondria with reduced fragmentation (Fig. 5E), accompanied by an increased MMP regardless of FGF21 treatment (Fig. 5F). Metabolic flux profiling demonstrated that, compared with FGF21 treatment alone, DRP1 knockdown attenuated the FGF21-induced increase in oxidative phosphorylation and glycolytic capacity following FCCP/oligomycin exposure (Fig. 5G, H). However, consistent with the lactate production data, the maximum level of glycolysis in DRP1-knockdown cells stimulated with FGF21 was still slightly elevated during the oligomycin challenge phase compared with that in DRP1-knockdown control cells, as shown in Fig.5H.

Collectively, these data establish that DRP1-driven mitochondrial fission enables FGF21’s tumor-promoting effects by mediating metabolic reprogramming.

### The CaMKII signaling pathway mediates FGF21-induced phosphorylation of DRP1 at Ser616 in LUSC

**Fig. 6:**
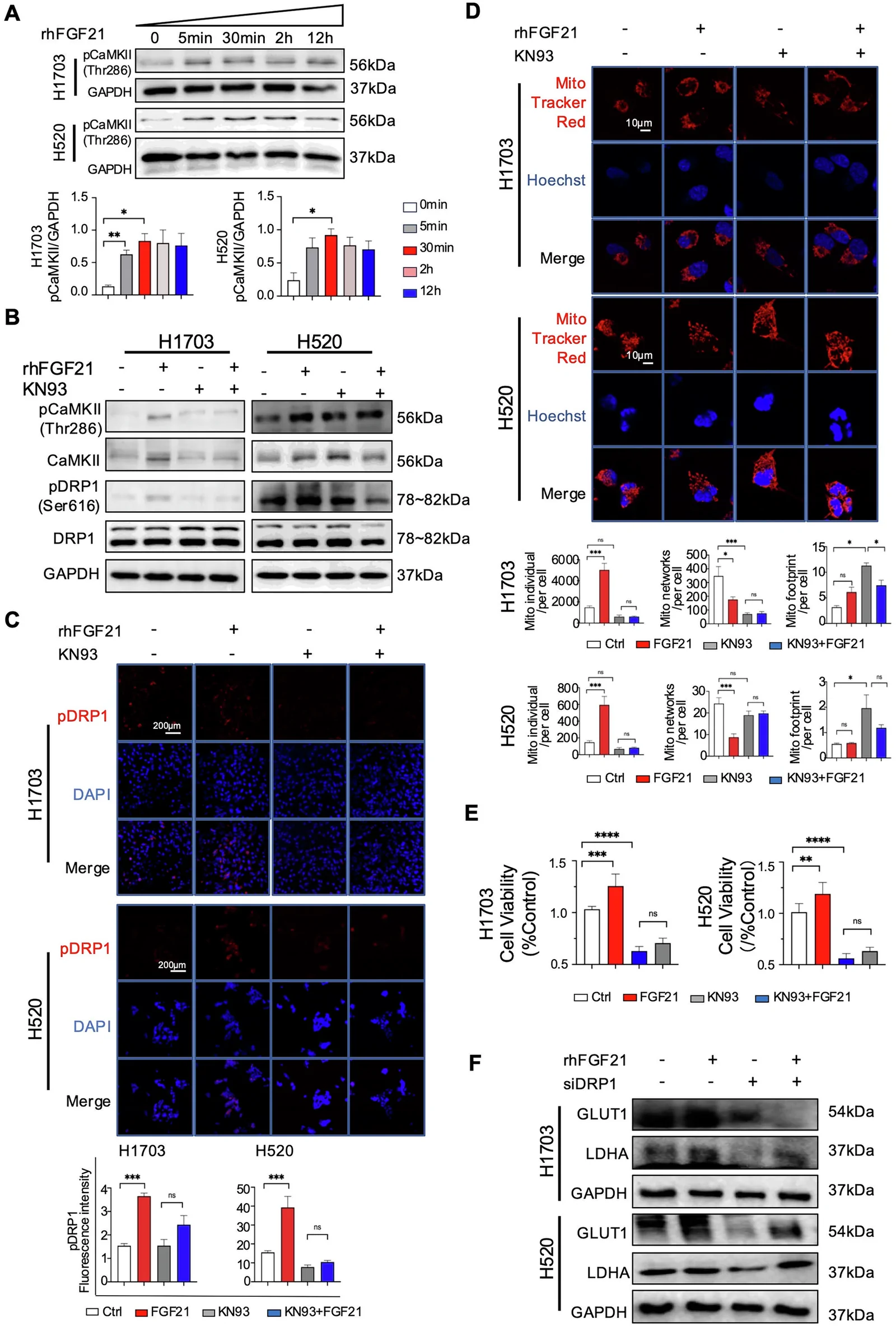
Inhibition of CaMKII blocks DRP1-mediated mitochondrial fission and tumor proliferation. **A** Representative western blotting images showing the protein expression of p-CaMKII (Thr286) in H1703 and H520 cells incubated with FGF21 for different durations. **B** Representative Western blotting images showing the protein expression of p-CaMKII (Thr286), CaMKII, p-DRP1 (Ser616) and DRP1 in H1703 and H520 cells incubated with FGF21 pretreated with KN93. **C** Immunofluorescence staining showing the expression and distribution of p-DRP1 (Ser616) and DRP1 in H1703 and H520 cells incubated with FGF21 pretreated with KN93. **D** Immunofluorescence staining showing the mitochondrial morphology in H1703 and H520 cells incubated with FGF21 pretreated with KN93. **E** CCK-8 assay showing the viability of H1703 and H520 cells incubated with FGF21 pretreated with or without KN93. **F** Representative western blotting images showing the expression of key glycolytic pathway components (GLUT1, LDHA) in DRP1-silenced LUSC cells with or without FGF21 stimulation. GAPDH served as a loading control. The data are shown as the means ± SEMs or SDs; *p < 0.05, **p < 0.01, ***p < 0.01, ****p < 0.01.

Given the essential role of DRP1 in FGF21 activation signaling, we investigated its upstream regulation. On the basis of established links between CaMKII and DRP1 [39], we propose that FGF21 activates this kinase. Immunoblotting at sequential timepoints revealed increases in phosphorylated CaMKII (p-CaMKII) in the FGF21-treated H1703/H520 cells, indicating signaling activation (Fig. 6A).

To validate the involvement of phosphorylated CaMKII (p-CaMKII) in FGF21-regulated mitochondrial processes in LUSC cells, pharmacological inhibition was performed using KN93, a selective CaMKII antagonist. Initial experiments assessed the inhibitory efficacy of KN93 on p-CaMKII levels under FGF21 stimulation, with immunoblotting data demonstrating significant suppression (Fig. 6B). Subsequent investigations revealed that CaMKII pathway inhibition substantially attenuated DRP1 phosphorylation (p-DRP1), as evidenced by Western blot analysis (Fig. 6B). Complementary immunofluorescence studies further confirmed these findings, revealing reduced p-DRP1 immunoreactivity following CaMKII blockade during FGF21 treatment (Fig. 6C). Consequently, KN93 pretreatment blocked FGF21-driven mitochondrial fission (Fig. 6D), demonstrating that CaMKII mediates DRP1 activation downstream of FGF21. We also assessed whether the inhibition of CaMKII influences the pro-proliferation effects of FGF21. The results showed that pretreatment with KN93 abolished FGF21-mediated enhancement of LUSC cells proliferation (Fig.6E).

Given that the FGF21-induced increases in lactate production and glycolytic capacity were not fully abolished by DRP1 silencing, we further examined the expression of key glycolysis-related proteins, including glucose transporter 1 (GLUT1) and lactate dehydrogenase A (LDHA). Our results revealed that the expression of these glycolytic markers were significantly upregulated during FGF21 treatment. Although DRP1 knockdown reduced their expression under FGF21 stimulation, this suppression was incomplete (Fig. 6F). These data indicate that FGF21 partially activates glycolysis through DRP1-dependent fission.

Taken together, these results demonstrated that FGF21 promoted DRP1 activation and mitochondrial fission in LUSC cells, which was dependent on the activation of CaMKII.

### In vivo FGF21 accelerates tumor growth and induces mitochondrial fission in LUSC xenografts

**Fig. 7:**
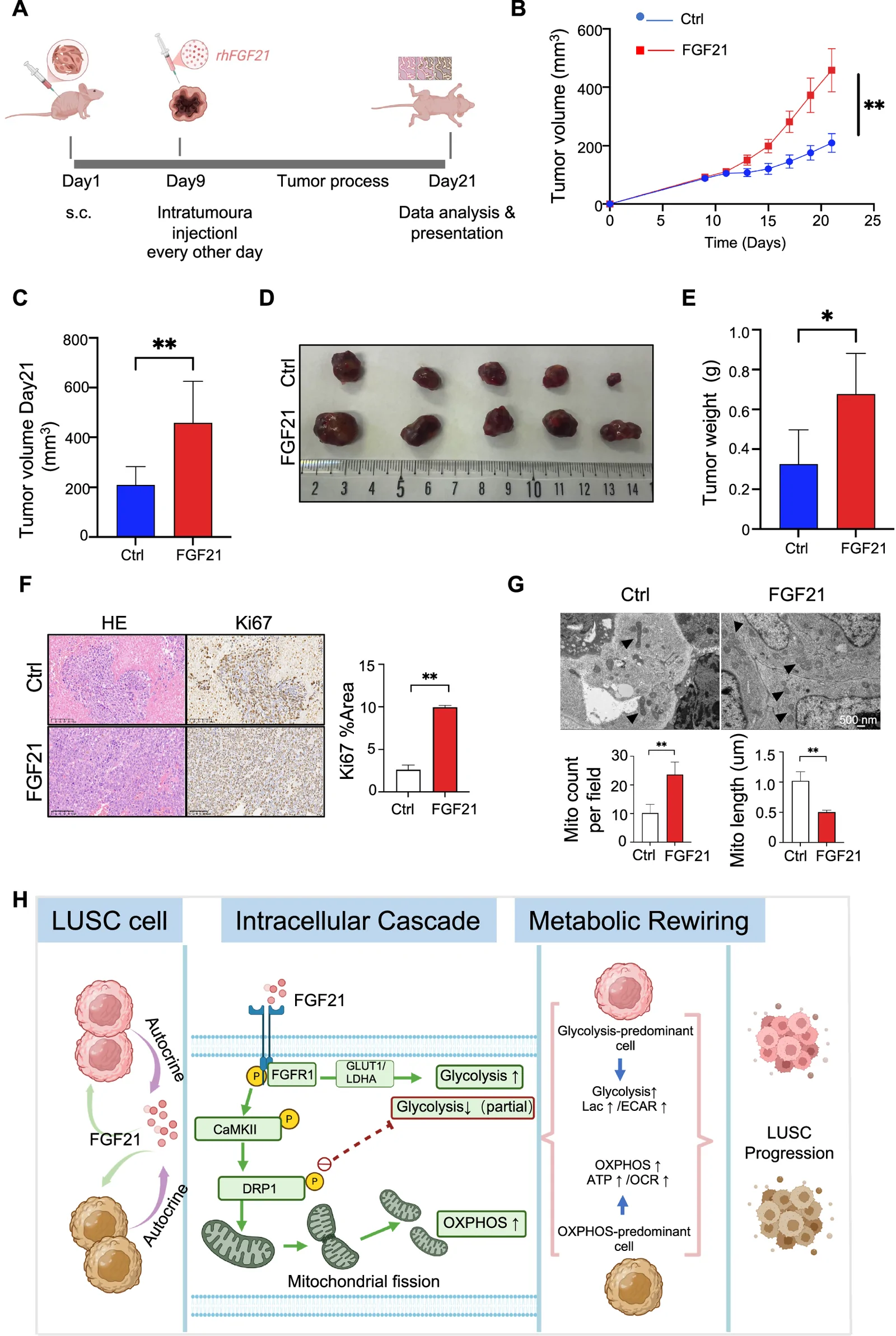
FGF21 promotes tumor growth and mitochondrial fission in LUSC xenograft models. **A** Experimental design. *s.c*., subcutaneous. **B**, **C** Tumor volumes were measured. **D** Representative images of dissected tumors from each treatment cohort. **E** Tumor masses were weighed at the endpoint. **F** Hematoxylin & eosin (H&E) staining and Ki-67 immunohistochemistry (brown nuclear staining) of xenograft sections; scale bars, 100 μm. **G** Representative images of mitochondrial morphology in tumor tissues using TEM. The black arrows indicate fragmented (rhFGF21 group) or tubular (control) mitochondria; scale bars, 200 nm. Mitochondrial counts per field (mean ± SEM, *n* = 5 fields at ×6000 magnification/group) and mitochondrial length (mean ± SEM, *n* = 8 mitochondria/group) were quantified using the TEM images. **H** Schematic illustration of how FGF21 orchestrates mitochondrial fission and metabolic reprogramming through the CaMKII/DRP1 axis in LUSC. Created in https://BioRender.com. The data are shown as the means ± SEMs or SDs; *p < 0.05, **p < 0.01, ***p < 0.01, ****p < 0.01.

To validate these findings in vitro, we established xenograft models in which H520 cells were implanted into mice. When the xenografts reached the standardized volume, the tumor-bearing mice were randomized into treatment cohorts (Fig. 7A). Administration of recombinant human FGF21 or the PBS control via intra-tumoral injection every other day was performed to maintain localized therapeutic concentrations. Compared with control xenografts, FGF21-treated xenografts presented accelerated growth curves (Fig. 7B), with obvious increases in tumor size and weight (Fig. 7C–E). It can therefore be concluded that FGF21 facilitates tumor proliferation in vivo.

Consistent with these findings, IHC revealed that FGF21 treatment increased the expression of Ki67 in xenograft tissues, corroborating the increased mitotic activity suggested by gross tumor metrics (Fig. 7F). Morphological changes in mitochondria were observed by TEM. The mitochondrial counts and length were calculated (Fig. 7G). The findings revealed that FGF21-treated tumor cells contained more mitochondria, while these mitochondria were smaller and rounder than those in the control cells.

These data indicated that FGF21 promotes LUSC proliferation in vivo. Additionally, FGF21 induces mitochondrial fission, leading to increased numbers of smaller mitochondria. Figure 7H shows the proposed mechanism by which FGF21 orchestrates mitochondrial fission and metabolic reprogramming through the CaMKII/DRP1 signaling axis.

## Discussion

This study revealed that FGF21 upregulation in LUSC tissues and cell lines promotes cancer cell proliferation, migration, and metabolic reprogramming through mitochondrial fission. Mechanistically, FGF21 activates CaMKII-mediated phosphorylation of DRP1 at Ser616, promoting mitochondrial fission and metabolism remodeling. These findings align with emerging evidence of the tumor-promoting functions of FGF21 in malignancies, including colorectal, breast, and melanoma, where it facilitates immune evasion via CD8 + T-cell metabolic rewiring [15, 16, 40–43]. However, reports of its tumor-suppressive function in pancreatic ductal adenocarcinoma (PDAC) reveal its paradoxical dual effects [19]. The differential effects of FGF21 on tumor growth may be tissue specific. First, the broad expression of FGFR1 in LUSC enables FGF21 to effectively trigger downstream signaling events. In contrast, PDAC tissue may lack the critical receptor components for FGF21-mediated tumor suppression. Second, pancreatic cancer progression is tightly linked to the inflammatory microenvironment, where FGF21 decreases pro-tumorigenic cytokine (e.g., IL-6) levels. In LUSC, FGF21 exerts cell-autonomous effects primarily on mitochondrial reprogramming rather than immunomodulation, as demonstrated in our study.

While FGF21 overexpression has been reported in hepatocellular, renal, and gastrointestinal malignancies [44–46], our investigation revealed that FGF21 operates through localized tumor-promoting mechanisms distinct from its classical endocrine functions [22]. Specifically, we observed pronounced FGF21 upregulation within tumor tissues, cellular models, and TCM, which was consistent with autocrine signaling activity, whereas circulating FGF21 levels were not significantly increased in patient sera. The inconsistency between high FGF21 levels in tumor tissue and unchanged serum FGF21 levels may occur because of two biological mechanisms. Local retention within the tumor microenvironment through extra matrix binding or receptor-mediated sequestration may limit systemic diffusion, whereas circulating FGF21 derived from multiple organs, including hepatic secretions, can mask tissue-specific expression patterns in serum biomarker analyses [23, 47]. Consequently, our in vitro and in vivo investigations focused on examining the local impact of FGF21 on LUSC cells or tumor tissues.

The observed inconsistency between bioinformatics analyses (linking FGF21 transcriptional expression to prolonged OS/PFI in LUSC) and our experimental demonstration of its localized pro-tumorigenic functions underscores the complex, context-dependent nature of FGF21 signaling in cancer. As reported in various studies, FGF21 can exhibit antitumor effects, protumor effects, and tumor immune evasion properties, indicating that its roles vary significantly across cell types, tumor stages, and tumor microenvironment conditions [16, 48], much like those of TGF-β [49]. Additionally, transcriptional profiles may poorly reflect the level of biologically active FGF21. The clinically observed protective effects may reflect systemic compensatory responses rather than direct tumor suppression. To resolve the conflicting survival data concerning FGF21 expression in LUSC patients, coculture models of tumor and immune cells may be needed in the future to investigate how tumor-secreted FGF21 modulates LUSC immunity. Moreover, quantification of FGF21 protein activation in situ through enriched longitudinal data analysis is needed.

Our work further revealed that the mitochondrial fission machinery is a previously uncharacterized downstream effector of FGFR signaling in LUSC. To meet cellular energy demands, mitochondria dynamically remodel their architecture through balanced fusion and fission events. In this study, we observed significantly elevated DRP1 expression in LUSC tissues compared with adjacent control tissues, mirroring reports on multiple cancer types [50–52]. DRP1 controls mitochondrial division through oligomerization and GTP hydrolysis at the outer mitochondrial membrane (OMM). Its activity is precisely regulated by opposing phosphorylation events: Ser616 phosphorylation promotes membrane fission, whereas Ser637 phosphorylation inhibits it [53]. In LUSC, FGF21 disrupts this balance by selectively promoting CaMKII-mediated DRP1 Ser616 phosphorylation and mitochondrial recruitment. This triggers mitochondrial fission without altering fusion regulators (MFN1/2, OPA1). Crucially, DRP1 silencing reversed fission phenotypes and abolished FGF21-driven proliferation, establishing it as the central mediator of the metabolic reprogramming. KN93-mediated CaMKII inhibition abrogated DRP1 Ser616 phosphorylation and mitochondrial fission, confirming the essential role of this kinase in this pathway, which is consistent with the knowledge of the regulatory functions of CaMKII [39]. These findings reveal a previously unreported FGFR1-CaMKII-DRP1 axis that operates beyond canonical PI3K/AKT or MAPK cascades typically associated with FGFR signaling [54]. While ERK1/2, a downstream effector of MAPK cascades, has been reported to phosphorylate DRP1 at Ser616 in other contexts [55, 56], the rapid kinetics of FGF21-induced mitochondrial fragmentation (within minutes) aligns more closely with the immediate Ca²⁺ flux and CaMKII auto-activation observed in other FGFR family members [57, 58]. This temporal distinction supports CaMKII as the primary proximal driver of this acute fission response in LUSC cells. Given that mitochondrial fission plays dual roles in generating new mitochondria and eliminating damaged components through distinct pathways (peripheral repair via Fis1 and midzone multiplication using the endoplasmic reticulum (ER)/actin/MFF protein) [59], further studies should examine DRP1 downstream events or more details of mitochondrial division patterns.

Contemporary research has revealed that malignant tumors exhibit metabolic plasticity, where OXPHOS and glycolysis frequently occur simultaneously rather than operating exclusively [60]. This metabolic plasticity is temporally regulated, where glycolysis predominantly drives rapid proliferation during local tumor expansion, whereas OXPHOS supports metastatic dissemination [60]. Our study demonstrates this principle in LUSC models with distinct origins. The primary-tumor-derived H520 line inherently exhibited greater glycolytic flux, whereas the metastatic-pleural-origin H1703 line maintained greater OXPHOS capacity. This baseline metabolic divergence was further validated by direct measurement of the OCR/ECAR ratio and analysis of glycolytic gene expression profiles from the CCLE database (Fig. S6). Correspondingly, FGF21 induced divergent metabolic reprogramming by promoting mitochondrial respiration in H1703 cells while promoting glycolysis in H520 cells. Critically, FGF21 elicited stronger proliferative responses in H520 cells than in H1703 cells, consistent with its higher endogenous expression in H520 and further reflecting differential cellular sensitivity. Notably, this expression pattern aligns with elevated FGF21 levels in M0 versus M1 LUSC tissues, suggesting intrinsic tumor cell properties may dictate FGF21-dependent oncogenic behaviors. However, the limited number of additional cell lines may restrict the broader application of our findings to the diverse subtypes of LUSC, and the mechanisms underlying the observed metabolic differences between cell types remain unclear. Furthermore, concurrent upregulation of GLUT1 and LDHA established a parallel pathway that enhances glycolytic flux, and this finding aligns with the conclusions of Wang, Li et al. [61]. FGF21 links these pathways to balance energy production and biosynthetic demands in tumors.

## Conclusion

This study identifies FGF21 as a tumor-promoting factor in LUSC that activates DRP1-mediated mitochondrial fission via CaMKII-dependent signaling. This process drives metabolic reprogramming including enhancement of glycolysis and oxidative phosphorylation, to support LUSC tumor progression. Cells respond differently to FGF21 on the basis of their inherent metabolic characteristics, leading to varied metabolic outcomes. Collectively, these findings establish the FGF21/ CaMKII/DRP1 pathway as a regulator of mitochondrial dynamics and metabolic adaptation in LUSC, providing potential therapeutic targets for LUSC treatment.

## Materials and methods

### Reagents and antibodies

The following small-molecule compounds and recombinant proteins were used: AZD4547 (S1476, Selleckchem); rhFGF21 (100-42, PeproTech; HY-P7012, MedChemExpress); and KN93 (HY-15465, MedChemExpress); Detailed information regarding all primary antibodies, secondary antibodies, and nuclear dyes (Hoechst33342 and DAPI) utilized in western blotting (WB), immunohistochemistry (IHC), and immunofluorescence (IF) is summarized in Table 1.

**Table 1:** List of primary antibodies, secondary antibodies, and nuclear dyes used in this study.

Antibody	Catalog no.	Host species	Source	Application (dilution)
Primary antibodies				
Anti-FGF21	3908	Rabbit	Abclonal	WB (1:3000); IHC (1:100)
Anti-FGFR1	9740	Rabbit	CST	WB (1:1000)
Anti-p-FGFR1 (Tyr653/654)	52928	Rabbit	CST	WB (1:1000)
Anti-OPA1	80471	Rabbit	CST	WB (1:1000)
Anti-MFF	84580	Rabbit	CST	WB (1:1000)
Anti-DRP1	8570	Rabbit	CST	WB (1:1000); IF (1:100)
Anti-DRP1	184248	Rabbit	Abcam	WB (1:1000); IHC (1:2000)
Anti-p-DRP1 (ser616)	4494	Rabbit	CST	WB (1:1000); IF (1:1000)
Anti-p-DRP1 (ser616)	137991	Rabbit	Absin	WB (1:1000)
Anti-p-DRP1 (ser637)	6319	Rabbit	CST	WB (1:1000)
Anti-MFN1	14739	Rabbit	CST	WB (1:1000)
Anti-MFN2	11925	Rabbit	CST	WB (1:1000)
Anti-TOM20	42406	Rabbit	CST	WB (1:1000); IF (1:200)
Anti-COXIV	4850	Rabbit	CST	WB (1:1000)
Anti-GLUT1	115730	Rabbit	Abcam	WB (1:100000); IF (1:100)
Anti-LDHA	52488	Rabbit	Abcam	WB (1:5000); IF (1:100)
Antibody	Catalog No.	Host Species	Source	Application (dilution)
Anti-CaMKII	5306	Mouse	Santa Cruz	WB (1:1000)
Anti-p-CaMKII (Thr286)	12716	Rabbit	CST	WB (1:1000)
Anti-GAPDH	2118	Rabbit	CST	WB (1:5000)
Secondary antibodies & dyes				
Goat anti-rabbit IgG	7074	Goat	CST	WB (1:3000)
Goat anti-rabbit IgG H&L (FITC conjungated)	E-AB-1014	Goat	Elabscience	IF (1:100)
AF647-labeled goat anti-rabbit IgG (H + L)	A0468	Goat	Beyotime	IF (1:200)
Hoechst33342	C0030		Solarbio	IF (as recommended)
DAPI	C0065		Solarbio	IF (As recommended)

*CST* cell signaling technology, *WB* Western blotting, *IHC* immunohistochemistry, *IF* immunofluorescence.

### Human specimens

Paired LUSC and histologically confirmed adjacent non-tumor tissues were collected from untreated patients during surgery.

Serum samples and associated individual data were collected from all participants in the morning before breakfast at Qilu Hospital (Qingdao), Shandong University. Serum was cryopreserved at −80 °C for subsequent analysis. The clinical and demographic characteristics associated were summarized in Table S1.

An ethically approved LUSC tissue array (HLugA150CS03, Outdo Biotech Co., Ltd., Shanghai, China) comprising 75 surgically resected tumor-normal pairs was also utilized. Associated characteristics were summarized in Table S3.

The study was approved by the medical ethics committee of Qilu Hospital (Qingdao) of Shandong University and followed the tenets of the Declaration of Helsinki (2018NL-106-02). Written informed consent was obtained from all study subjects.

### ELISA

Serum levels of soluble FGF21 were measured using a commercial ELISA kit (ABclonal Biotechnology Co., Ltd) following the manufacturer’s protocol. The concentration of FGF21 was interpolated from a standard curve generated using the respective recombinant protein.

### Cell lines and cell culture

The human lung squamous carcinoma cell lines, H1703 and H520, were sourced from Wuhan Pricella Biotechnology Co., Ltd. These cells were routinely cultured in RPMI-1640 medium with 10% fetal bovine serum (FBS) and antibiotics at 37 °C with 5% CO_2_. The human normal bronchial epithelial cell line BEAS-2B was kindly provided by Dr. Jiajia Qu (Shandong University) and maintained in DMEM medium (Life Technologies) supplemented with 10% FBS (HyClone) and antibiotics at 37 °C with 5% CO_2_. Cells were starved overnight and cultured in 3% FBS medium incubated with rhFGF21 or pretreated with KN93, AZD4547.

### Cell functions in vitro

Cell’s viability was determined by the CCK-8 assay (Boster, Wuhan, China) according to the manufacturer’s protocols. 2000 cells/well were seeded in 96-well plates and treated with different concentrations of rhFGF21 (0, 25, 50, 200 ng/ml). After 24, 48, 72 hours, cells viability was measured by addition of 10 μl of CCK-8 solution. The absorbance of optical density (OD) values of each well were read and recorded at 450 nm with a microplate reader. Each group were performed in triplicate.

Cell’s proliferation was determined by colony formation assay. Cells were plated in six-plates of 500 cells/well, and cultured for 10–14 days of incubation. There were three replicate wells in each group. After fixation by 4% paraformaldehyde (20 min), cells were stained with Giemsa dye solution (10–20 min), rinsed with PBS, and air-dried. Colonies were then photographed and quantified to assess treatment effects.

Cell’s migration ability was determined by the wound healing assay. Cells were plated in six-well plates to 90% confluence. After 12 h, a uniform scratch was created in the monolayer. Cells were maintained in 3% FBS medium containing either rhFGF21 or control. Wound closure was monitored at 12, 24, and 48 h using an Olympus inverted microscope (Beijing). Migration rates were calculated as: [(Wound width at 0 h–Wound width at 24 h) treated/(Wound width at 0 h–Wound width at 24 h) control] × 100.

Cell invasion ability was determined by the transwell assay. First, invasion chambers were thawed from −20 °C and allowed to equilibrate to room temperature on a sterile surface. Following this, both the upper and lower chambers were pre-hydrated with 500 μL serum-free medium before being transferred to a 37 °C incubator for 2 hours to activate the Matrigel matrix. Subsequently, a serum-free cell suspension containing 1 × 10^5^ cells was carefully seeded into each upper chamber to initiate the invasion assay. The 3% FBS medium in the presence or absence of rhFGF21 was added to the lower chamber. Cells were stained with Giemsa solution, and invading cells were counted by photographing the membrane under a microscope.

Tumour sphere assays were generated and expanded in Basal Medium supplemented with B-27 and bFGF (HUXUC-90062, OriCell). 1000 cells/ml were seeded in ultra-low attachment plates (Corning) as described previously [62]. For serial passaging, spheres were collected after 7 days using a 40 mm cell strainer, dissociated into single cells with trypsin, and then re-cultured under identical conditions for another 7 days with or without rhFGF21. During this process, spheres larger than 40 mm were enumerated to assess growth effects.

### Cell transfections

For siRNA transfection, cells were plated in 6-well plates and cultured until 70% confluent. Next, three distinct DRP1-targeting siRNAs and a negative control siRNA were introduced using Lipofectamine 2000 (Invitrogen, Carlsbad, CA), following the manufacturer’s instructions. After 48 h, transfected cells were isolated, and knockdown efficiency was verified by Western blot. The siRNA sequences used were as follows:

siRNA1 (si1-DRP1): Sense: 5′-GGAGCCAGCUAGAUAUUAATT-3′ Antisense: 5′-UUAAUAUCUAGCUGGCUCCTT-3′

siRNA2 (si2- DRP1): Sense: 5′-GGUGCUAGAAUUUGUUAUATT-3′ Antisense: 5′-UAUAACAAAUUCUAGCACCTT-3′

siRNA3 (si3- DRP1): Sense: 5′-GGCCAGCUGUAUAAAUCAUTT-3′ Antisense: 5′-AUGAUUUAUACAGCUGGCCTT-3′

siRNA control (si-ctrl): Sense: 5′-UUCUCCGAACGUGUCACGUTT-3′ Antisense: 5′-ACGUGACACGUUCGGAGAATT-3′

### Cytoplasmic and mitochondrial protein extraction

H1703 and H520 cells were collected from cultures treated with or without rhFGF21, washed with phosphate-buffered saline (PBS), and pelleted by centrifugation (600 × *g*, 5 min, 4 °C). The cell pellets were resuspended in mitochondrial isolation buffer (C3601, Beyotime) for 15 min on ice, followed by gentle homogenization using a glass homogenizer. After low-speed centrifugation (600 × *g*, 10 min, 4 °C) to remove debris, the supernatant was further centrifuged (12,000 × *g*, 10 min, 4 °C) to separate mitochondrial (pellet) and cytoplasmic (supernatant) fractions. Isolated mitochondria were lysed in buffer containing 1% PMSF for 30 min to extract proteins for western blot analysis.

### Conditioned medium (CM)/tumor-conditioned medium (TCM) protein extraction

Based on prior described [37], cells were maintained in serum-free medium within multi-well plates. Supernatants are harvested by gentle aspiration and pooled by replicate group to minimize variability. Cellular debris is removed by centrifugation at 1000 × *g* for 5 minutes at room temperature. For protein concentration, clarified supernatants undergo methanol-chloroform precipitation: to 500 μL supernatant, add 500 μL methanol followed by 125 μL chloroform, then vortex vigorously for ≥30 seconds until a homogenous milky suspension forms. Centrifuge at 13,000 × *g* for 5 minutes to separate phases (aqueous top, protein interphase, organic bottom). Carefully remove the aqueous phase by suction without disturbing the protein layer. Add 500 μL methanol to the retained organic phase and protein interphase, vortex to disrupt the protein layer, and re-centrifuge at 13,000 × *g* for 5 minutes to pellet precipitated proteins. Discard methanol supernatant, air-dry pellets briefly at 55 °C until translucent, then resuspend in SDS sample buffer. After vigorous vortexing and brief centrifugation, denature samples at 95 °C for 5 minutes prior to Western blot analysis. Processed samples are stored at −20 °C if not immediately analyzed.

### Western blot analysis

Cells were lysed in pre-cooled radioimmunoprecipitation (RIPA) buffer (P0013C, Beyotime) with protease/phosphatase inhibitors. Protein concentration was quantified using a BCA kit (P0012, Beyotime). Lysates (20 µg) were denatured, separated on 10–12% SDS-PAGE, and transferred to membranes. Membranes were blocked with 5% non-fat milk, incubated with primary antibodies overnight at 4 °C, then with HRP-conjugated secondary antibodies (1:5000–10,000) for 1 h. Bands were visualized using enhanced chemiluminescence (ECL) and analyzed with Fiji software.

### Immunofluorescent (IF) staining

Samples were fixed with 4% paraformaldehyde (15 min, RT) after PBS washing, followed by three 5-min PBS rinses. Permeabilization was performed using 0.1% Triton X-100 (10 min, RT), and non-specific binding was blocked with 3% BSA (1 h, RT). After PBS washing, samples were incubated overnight at 4 °C with primary antibody diluted in 3% bovine serum albumin (BSA). Excess antibodies were removed by PBS rinsing, and samples were exposed to pre-adsorbed secondary antibody (1 h, dark). Nuclei were counterstained with DAPI or Hoechst 33342. Imaging was performed on a Leica confocal microscope, with three random fields captured per sample.

### Transmission electron microscope (TEM)

Following fixation in 2.5% glutaraldehyde, tumor tissues were post-fixed with 1% osmium tetroxide, then dehydrated and embedded. Ultrathin sections were prepared, stained, and examined using a Hitachi transmission electron microscope at ×6000 and ×20,000 magnifications.

### Respirometry analysis and extracellular flux analysis

LUSC cells were seeded in XF24 microplates and cultured overnight in growth medium (37 °C, 5% CO_2_). Before analysis, cells were rinsed twice with assay medium (Seahorse XF Base Medium containing 2 mM L-glutamine, 1 mM pyruvate, and 10 mM glucose), followed by a 30-minute equilibration in a non-CO_2_ incubator at 37 °C. Mitochondrial respiration and glycolysis were measured using a Seahorse XF24 Analyzer (Agilent Technologies). Following baseline recordings, metabolic inhibitors/activators were sequentially injected to assess real-time changes in oxygen consumption rate (OCR) and extracellular acidification rate (ECAR). Data were normalized to total protein and processed with Wave software (Agilent).

### Measurement of mitochondrial membrane potential (MMP)

Mitochondrial membrane potential was assessed using JC-1 dye (C2006, Beyotime Biotechnology). Cells were seeded in six-well plates at a density of 3 × 10^5^/well and cultured in 5% CO_2_ at 37 °C. The JC-1 working solution (prepared by mixing 900 μL sterilized deionized water, 2 μL 500×JC-1 stock, and 100 μL 10× incubation buffer) assed to cells and incubated for 20 min (5% CO_2_ at 37 °C). Cells were then centrifuged at 350 × *g* for 5 min, washed twice with 1× incubation buffer, and resuspended in 500 μL 1× incubation buffer. Fluorescence intensity ratios (529 nm/590 nm) of JC-1 monomers to aggregates were quantified using a fluorescence microscope.

### Detection of ATP and lactate measurement

#### ATP quantification

LUSC cells (1 × 10^5^/well) under various treatment conditions were plated in six-well plates and cultured overnight. Total ATP levels were then determined using the ATP Chemiluminescence Assay Kit (E-BC-F002, Elabscience), with measurements normalized to cell count.

#### Lactate production assay

LUSC cells (6 × 10^5^/well) under various treatment conditions were cultured in six-well plates for 48 hours. Conditioned medium was collected and analyzed for lactate content using the L-Lactate Assay Kit (E-BC-K044-M, Elabscience). All values were normalized to total cellular protein.

### Analysis of mitochondrial morphology in the cells

Cells (4 × 10^5^/35-mm plate) on coverslips were fixed with 4% PFA, permeabilized, and stained with anti-tom20 antibody followed by Cy3-conjugated secondary IgG. Nuclei were visualized with DAPI or Hoechst33342. For live-cell imaging, mitochondria were labeled with Mito Tracker Red (C1049B, Beyotime, 100 nM, 37 °C, 30 min) or Mito Tracker Green (C1048, Beyotime) and imaged using a confocal microscope (Leica, ×60 oil objective). To avoid selection bias during image acquisition, fields of view were randomly selected based on the nuclear DAPI/Hoechst channel before switching to the mitochondrial channel. Confocal images were acquired from ≥3 randomly chosen fields per group (5–13 images per coverslip). Binary and skeletonized mitochondrial images were generated using Fiji ImageJ. For unbiased quantification, all image files were coded and analyzed by an investigator blinded to the experimental treatment groups. Mitochondrial branch length and number were quantified using the Mitochondrial Network Analysis (MiNA) toolset in Fiji. For each independent experiment, 10 individual cells were randomly selected and analyzed per condition, and data were collected from three independent biological replicates (total of 30 cells per condition).

### Subcutaneous human xenografts

All procedures were conducted in compliance with protocols approved by the Animal Ethics Committee of Qilu Hospital (Qingdao), Shandong University (Approval No. KYDWLL-202437). Xenograft models were generated by subcutaneous injection of 1 × 10^6^ H520 cells into the right flank of 5-week-old male BALB/c nude mice (Jiangsu HuaChuangXinNuo Medical Technology Co., Ltd.). Tumor growth was monitored every two days using the formula (length × width²)/2.

Once tumors reached 50–100 mm³, mice were randomized into control (PBS) and treatment (1 μg/mouse rhFGF21) groups (*n* = 5/groups). Treatments were administered via multipoint intratumoral injections every 48 hours. The study endpoint was set at a tumor volume of ~1000 mm³, at which point mice were euthanized for tissue collection.

### Histological analysis

Lung squamous carcinoma and xenograft tissues were analyzed for FGF21, DRP1 and Ki-67 expression, respectively. Following fixation in 10% neutral buffered formalin and paraffin embedding, sections were prepared and deparaffinized. Antigen retrieval was performed by microwave heating in 10 mM citrate buffer for 30 minutes after endogenous peroxidase blocking with 3% H_2_O_2_ (10 minutes). Primary antibodies were applied and incubated overnight at 4 °C. After PBS washes, sections were treated with secondary antibody, developed with DAB substrate, counterstained with hematoxylin, and mounted for microscopic evaluation.

### Bioinformatics and statistical analysis

Pan-Cancer Profiling of FGF21 mRNA Expression and Clinical Correlation in LUSC Differential FGF21 mRNA expression between tumor tissues and adjacent normal tissues across cancer types was analyzed using TCGA data. Further evaluation of FGF21 expression correlation with clinicopathological parameters in LUSC was conducted via R software. Results were visualized using ggplot2 and pheatmap packages.

Gene Ontology (GO) and Kyoto Encyclopedia of Genes and Genomes (KEGG) Analysis Transcriptomic data (raw counts and fragments per kilobase of transcript per million mapper reads (FPKM)) for TCGA-LUSC cohorts were acquired from the Genomic Data Commons portal (https://portal.gdc.cancer.gov/). Using the clusterProfiler R package, we performed Gene Ontology and KEGG pathway assessments with significance thresholds of false discovery rate (FDR) < 0.25 and nominal *p* < 0.05. Resultant pathway enrichments were visualized through specialized plots.

Association Between FGF21 Expression and Tumor Immune Infiltration in LUSC. The CIBERSORT algorithm in R was employed to quantify tumor-infiltrating immune cells, including adaptive/innate immune subsets (B cells, CD8 + T cells, NK cells, macrophages, dendritic cells) and specialized T-cell populations (Th1, Th2, Treg, Tfh, γδ T cells). Correlation analyses between FGF21 expression and immune cell abundance were assessed via Spearman’s rank test. Additionally, associations with immune cell-specific gene markers were evaluated, including:

T cell markers: CCR1-6/8, CD8A/B, CXCR3/5, HAVCR1, ICOS, FOXP3, IL2R/7 R/21 R/23 R, IL12RB1, NT5E

Macrophage markers: CD68, CD163, CD209, CD86, CCL2, IRF5, MRC1, NOS2, PTGS2.

Heatmaps and scatterplots visualized significant correlations (*p* < 0.05).

### FGF21 co-expression networks in mitochondrial dynamics and glycolysis

Pearson correlation analysis identified genes co-expressed with FGF21 in TCGA-LUSC, focusing on:

Mitochondrial dynamics: DNM1L, MFN1/2, OPA1, FIS1

Glycolysis: HK1/2, PKM, LDHA/B, SLC2A1-3, ENO1, G6PD, PDHB, PDK3, PDK4, PGK1, PKM, SLC2A1-3.

Using TCGA-LUSC transcriptomic data, we computed Pearson correlation coefficients to evaluate associations between FGF21 and candidate mRNA expression levels. Resulting correlations were visualized to identify significant relationships.

### Statistical analysis

Data are presented as mean ± SD/SEM. Intergroup comparisons used Student’s two-tailed *t* test for two groups, while multi-group analyses employed one-way ANOVA with post-hoc testing via GraphPad Prism 9.0. Statistical significance was defined as *p < 0.05, **p < 0.01, ***p < 0.001, and ****p < 0.0001.

## Supplementary information

Supplement Figure Legend

Figure S1

Figure S2

Figure S3

Figure S4

Figure S5

Figure S6

Table S1

Table S2

Table S3

Language editing certification

Raw images of Western blots

## Data Availability

All data generated or analyzed during the study are available.
