# Supplementary material for: Dual-faced metabolic reprogramming powers proliferation: FGF21 drives lung squamous carcinoma progression via CaMKII-DRP1-mediated mitochondrial fission
Source: Cell Death Dis. 2026 Jul 16;17(1):877. doi: 10.1038/s41419-026-09084-3 (PMC13655037; doi:10.1038/s41419-026-09084-3)

Original full length western blots

Fig.1E

FGF21 GAPDH

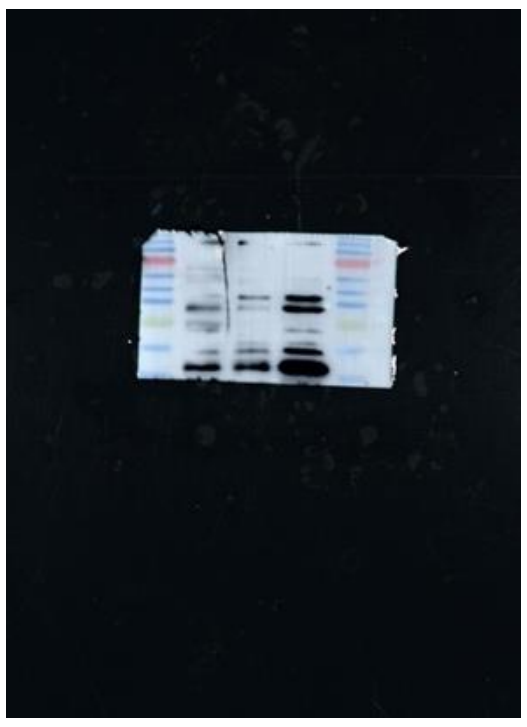

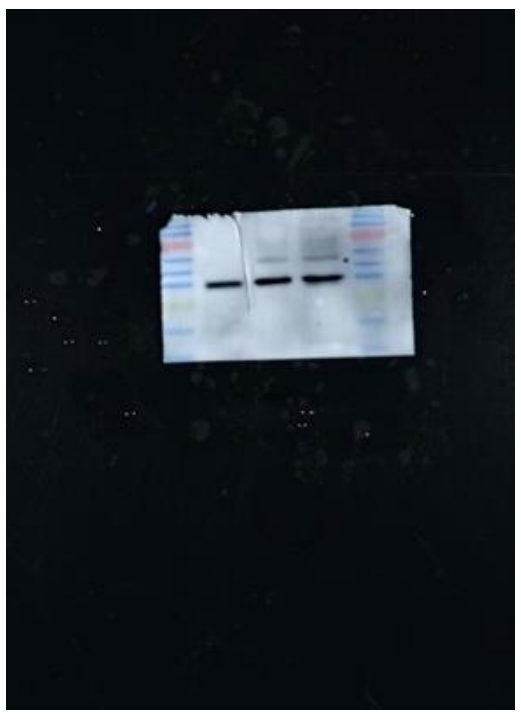

Fig.1F

FGF21

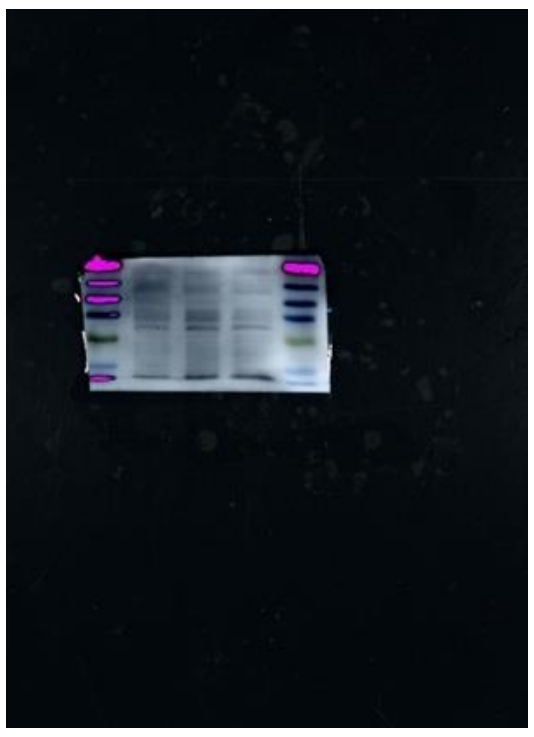

Fig.4A

pDRP1(Ser616)

H1703 H520

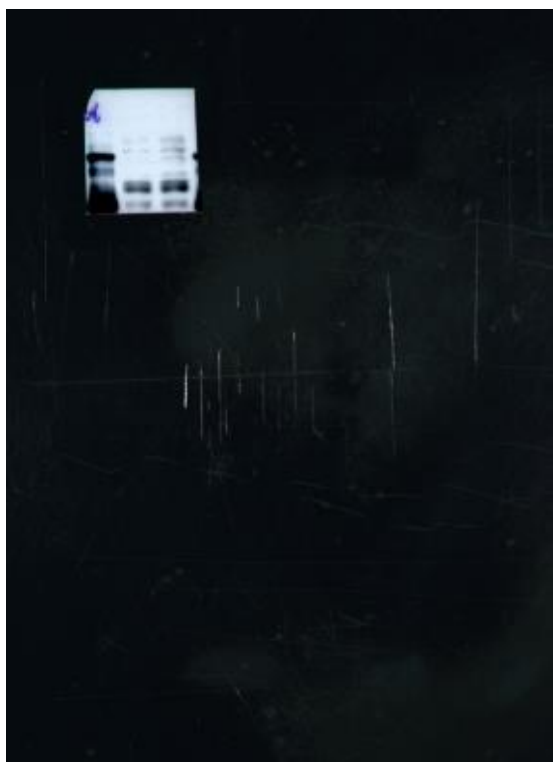

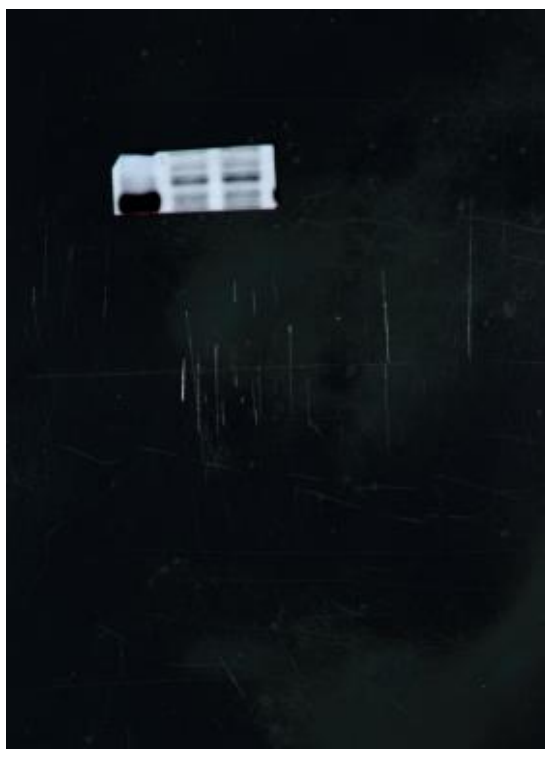

pDRP1(Ser637)

H1703 H520

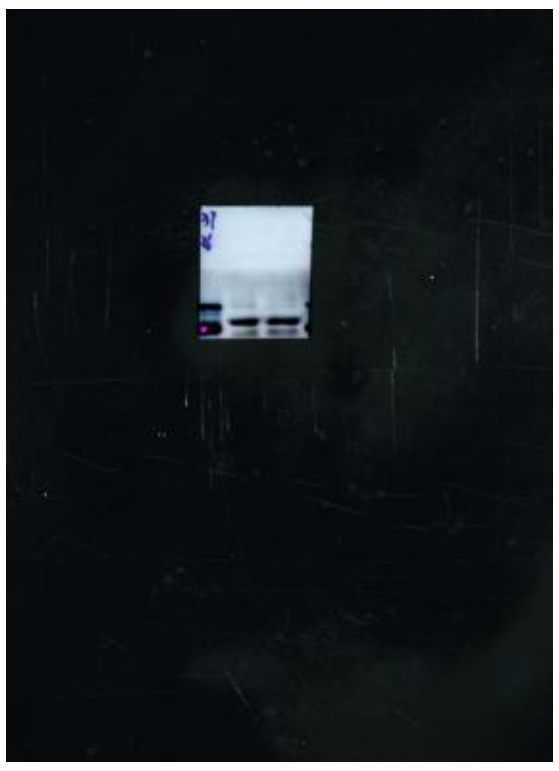

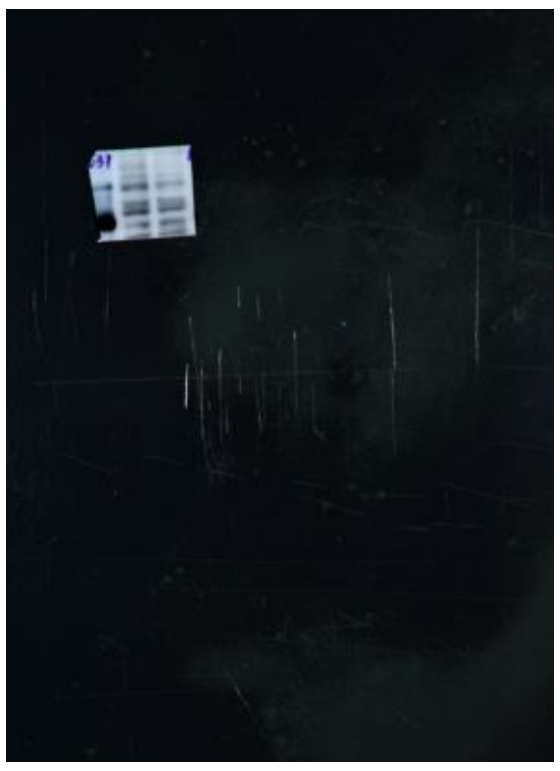

DRP1

H1703 H520

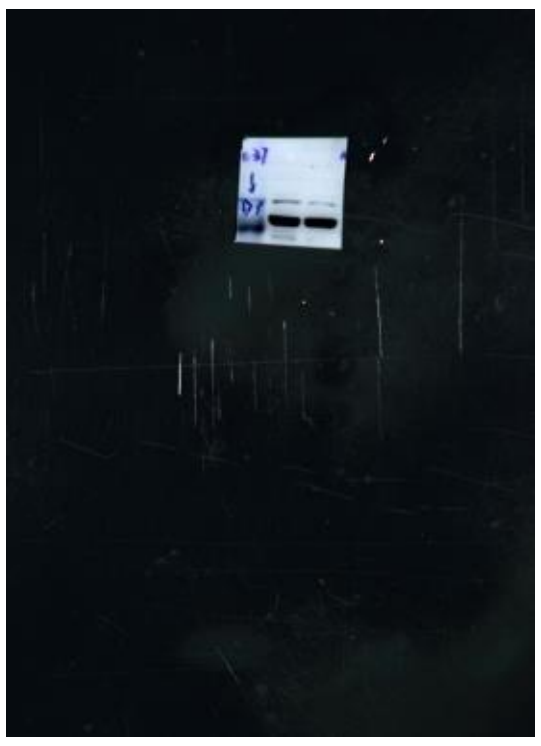

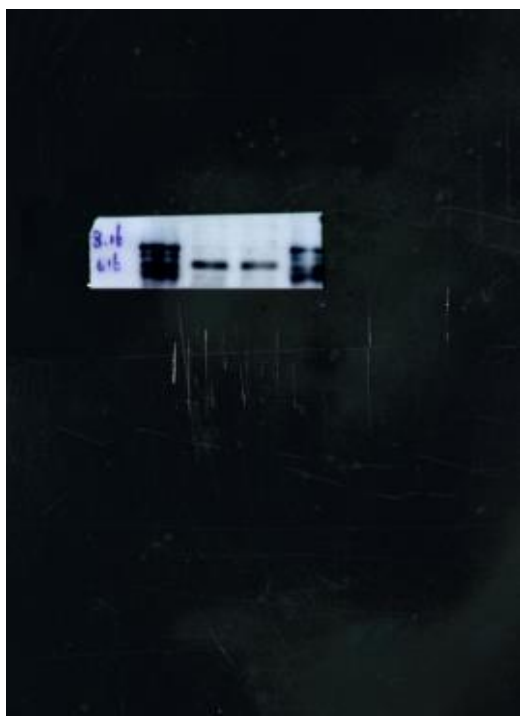

MFN2

H1703 H520

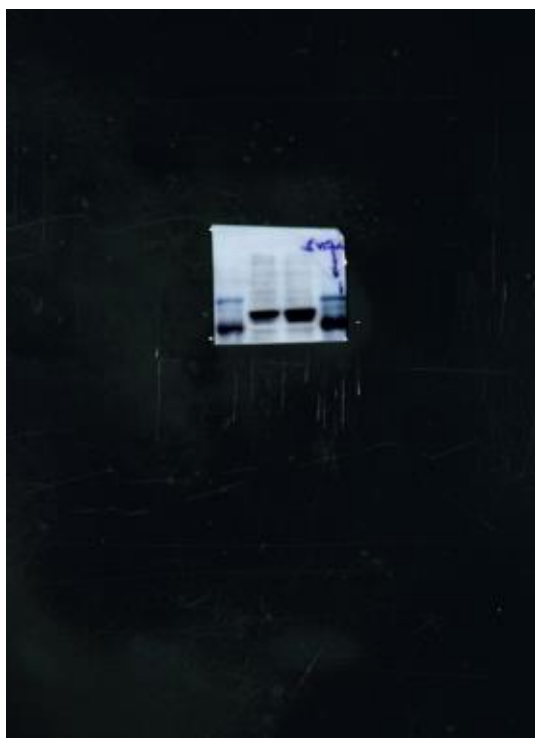

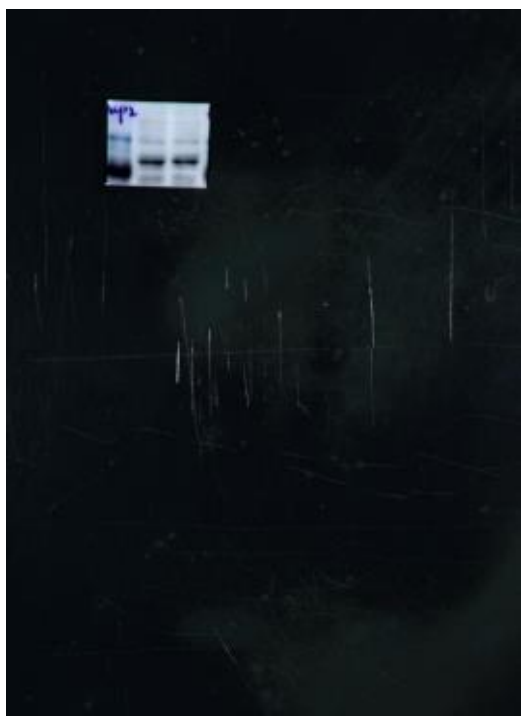

MFN1

H1703 H520

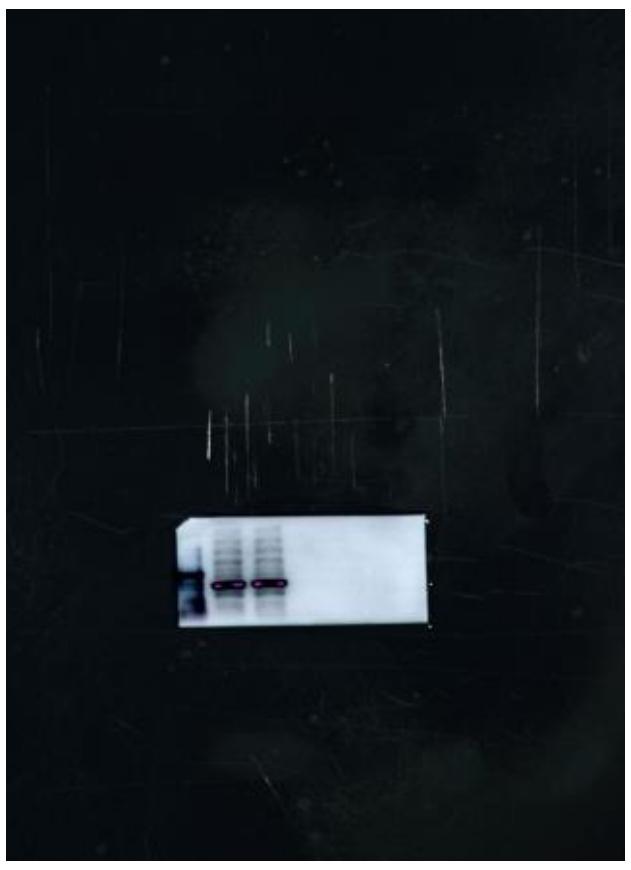 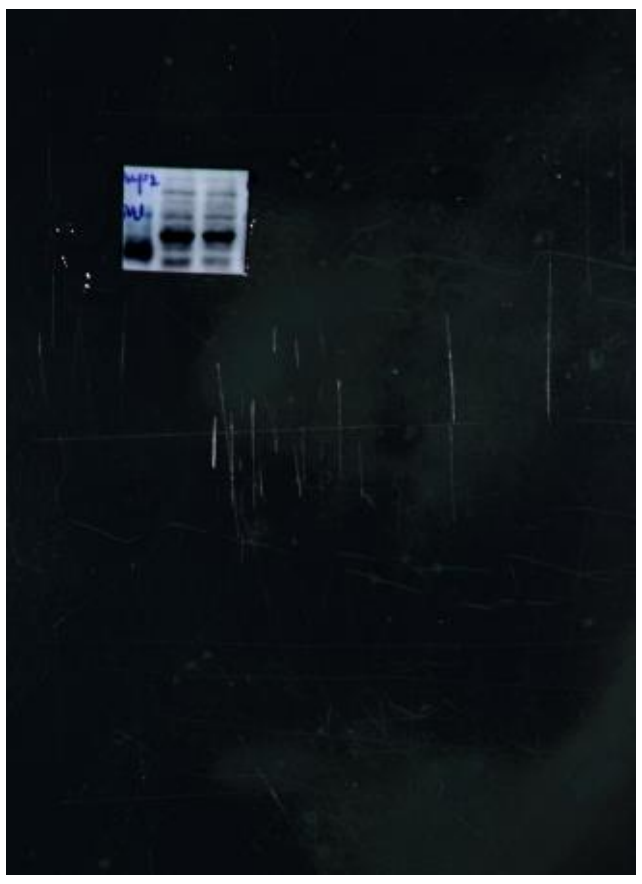

OPA1(L+S)

H1703 H520

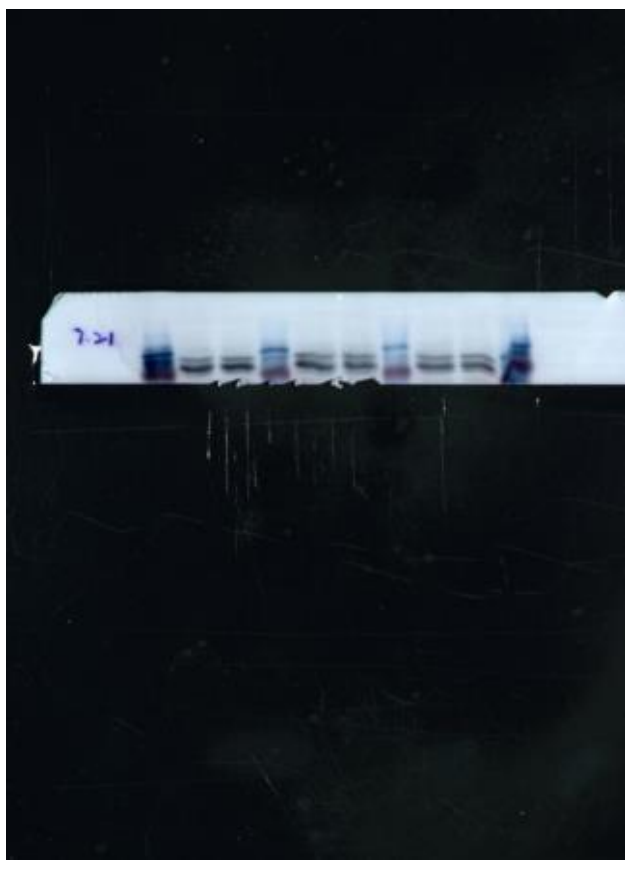 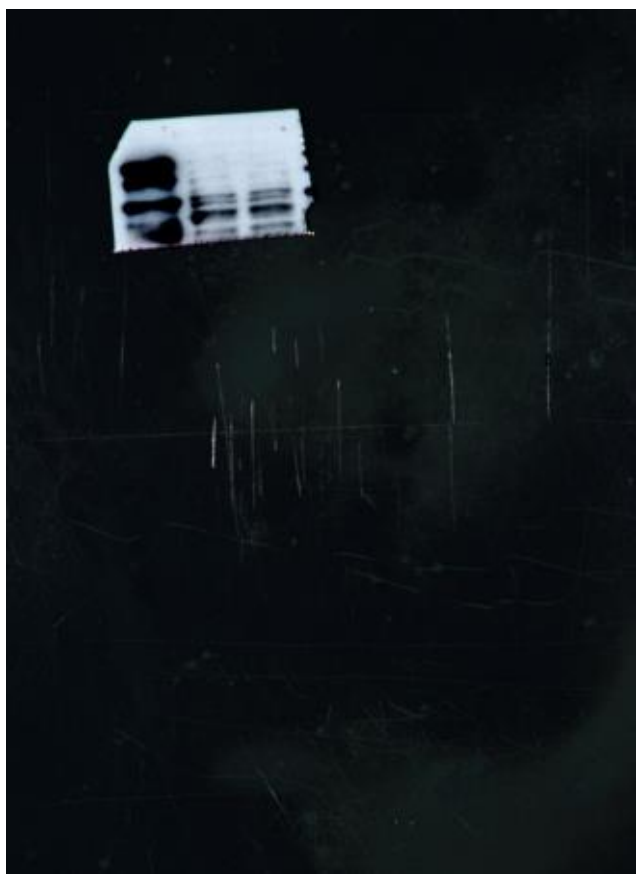

MFF

H1703 H520

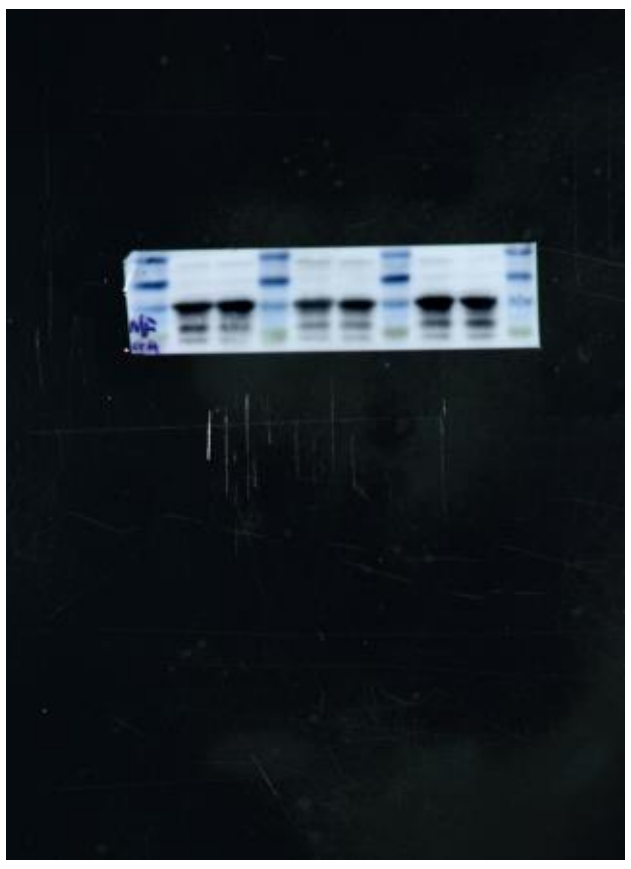 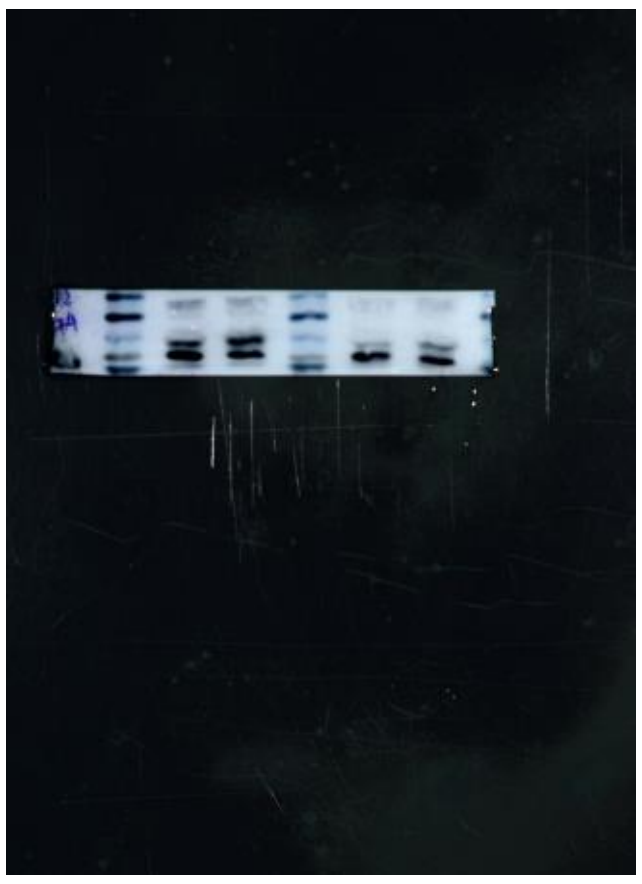

GAPDH

H1703 H520

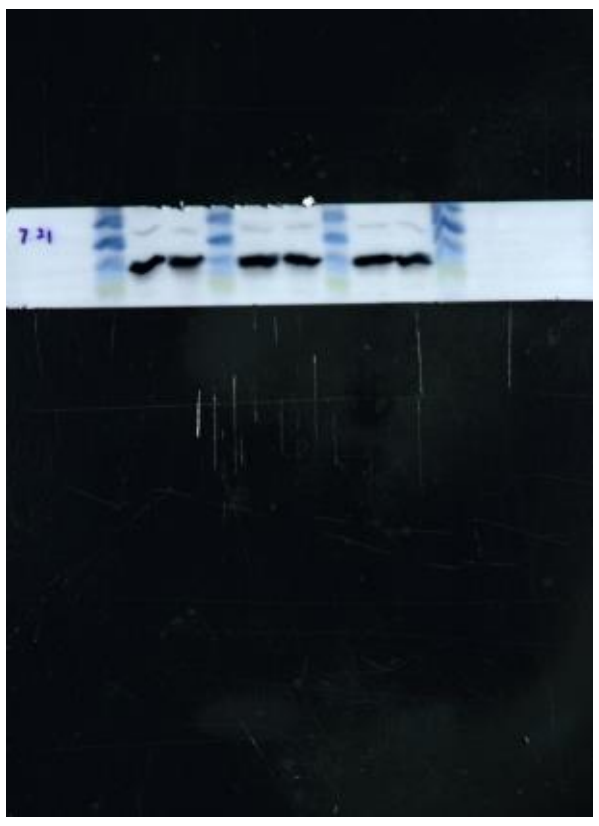

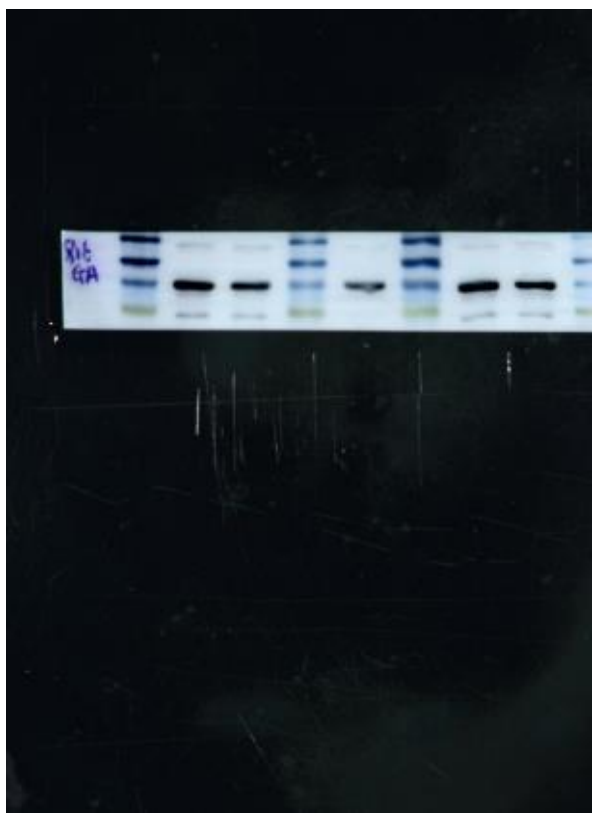

Fig.4B

pDRP1(Ser616)

H1703 H520

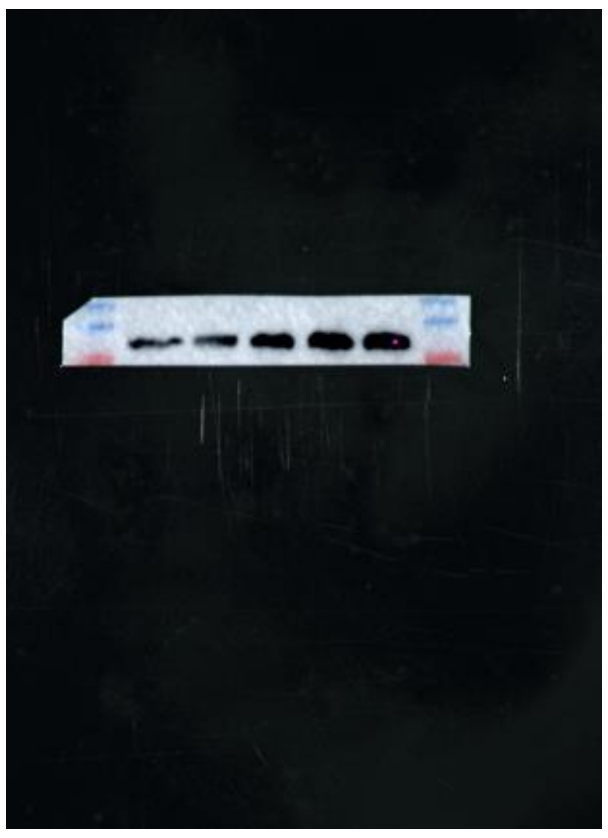

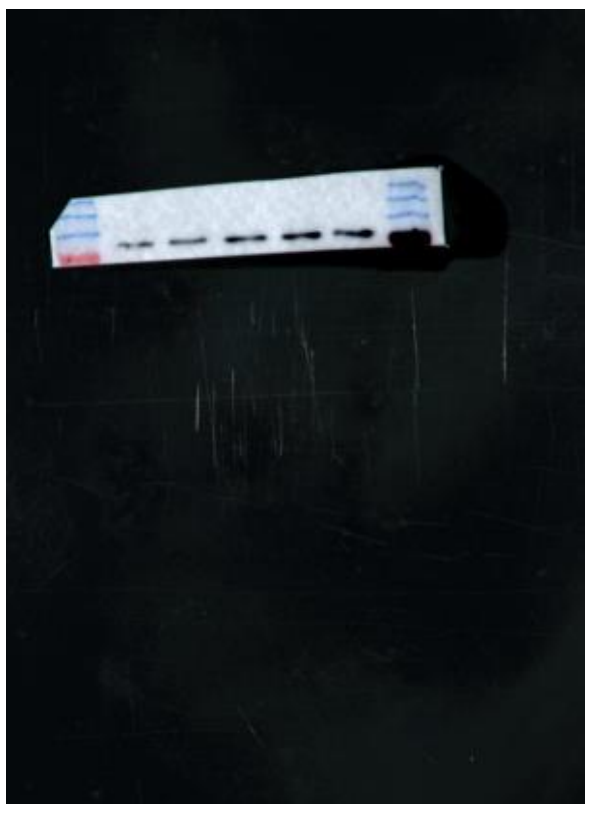

DRP1

H1703 H520

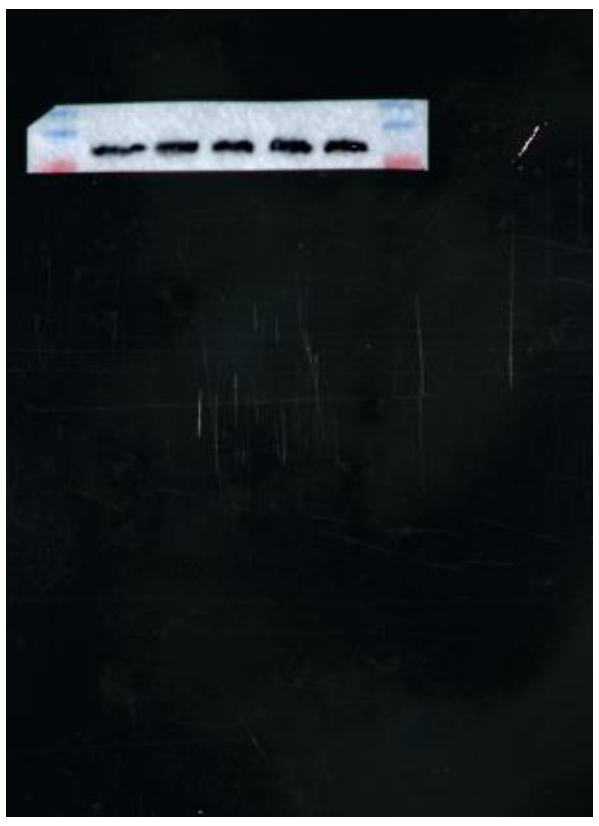

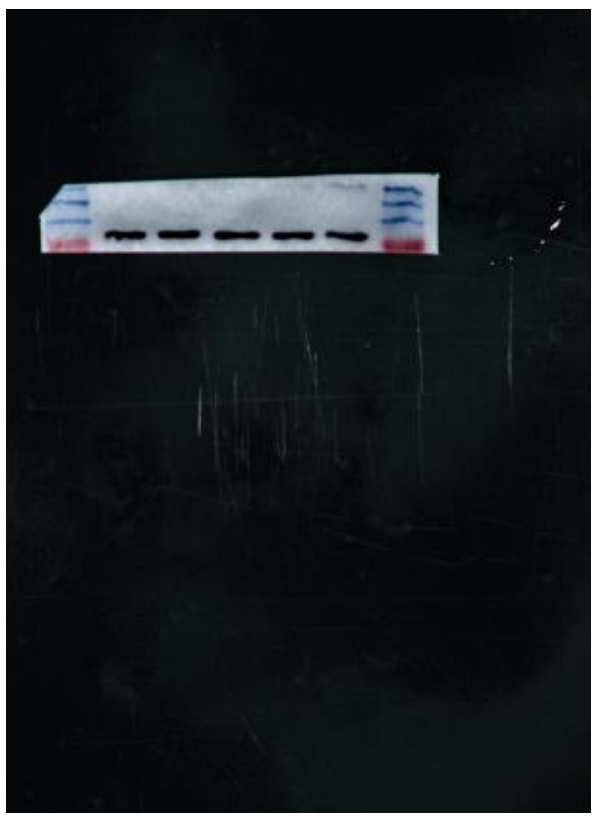

GAPDH

H1703 H520

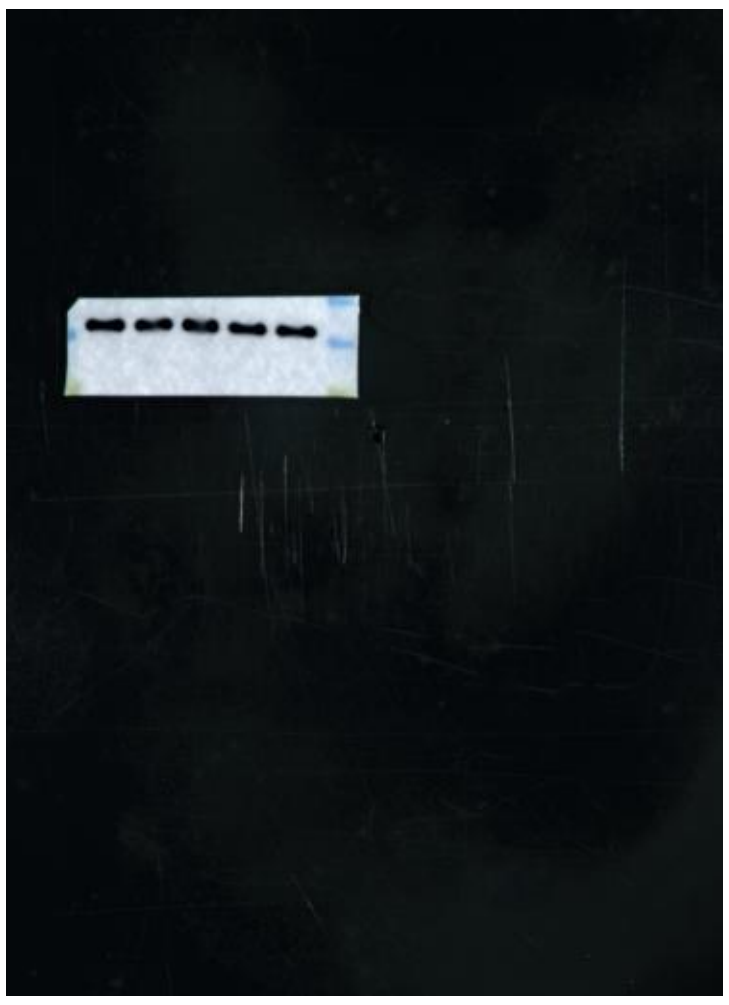

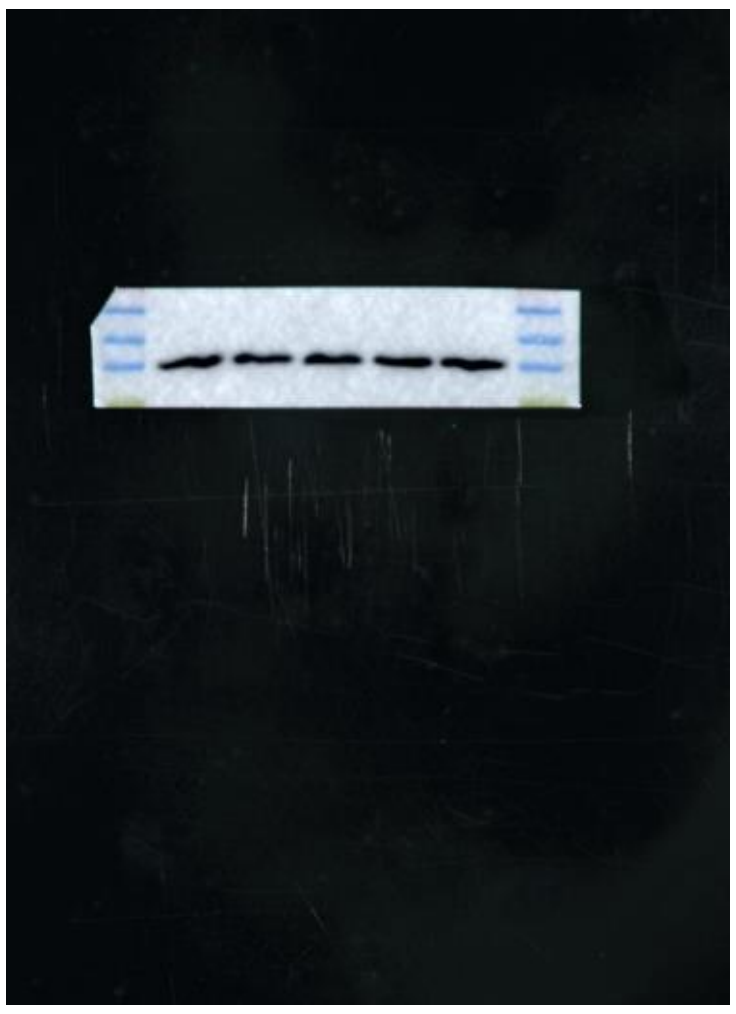

Fig.4D

H1703

DRP1

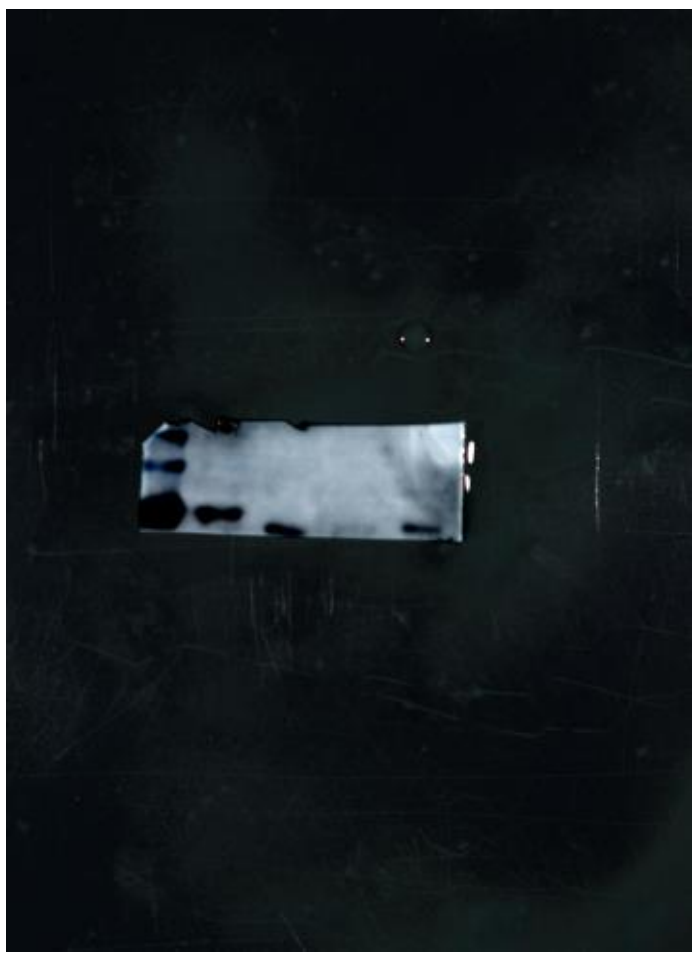

COX-IV

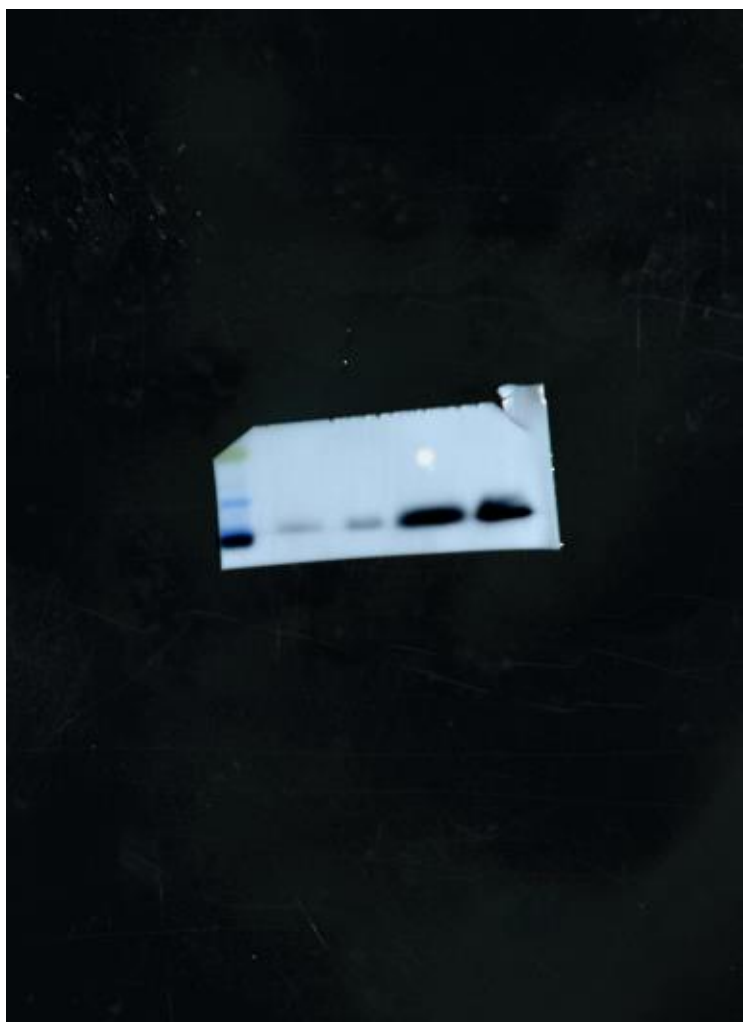

β-actin

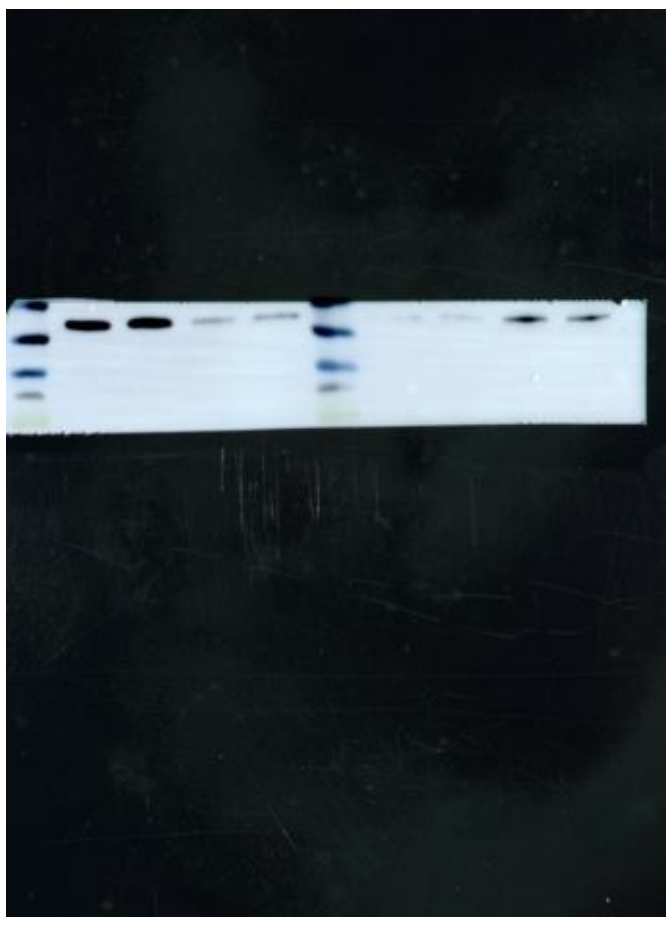

H520

DRP1

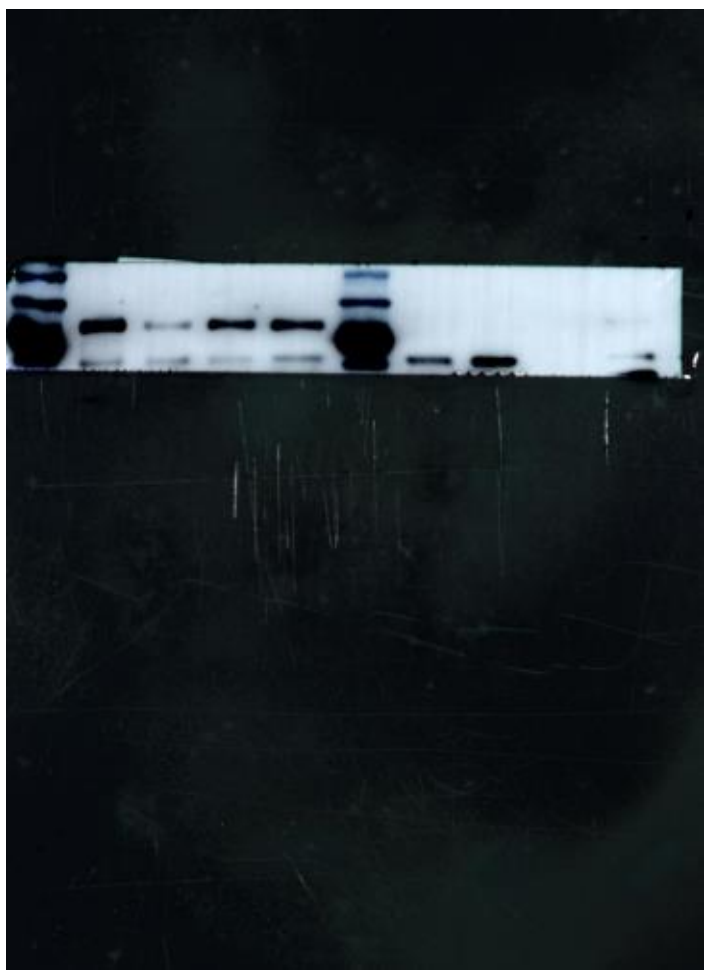

COX-IV

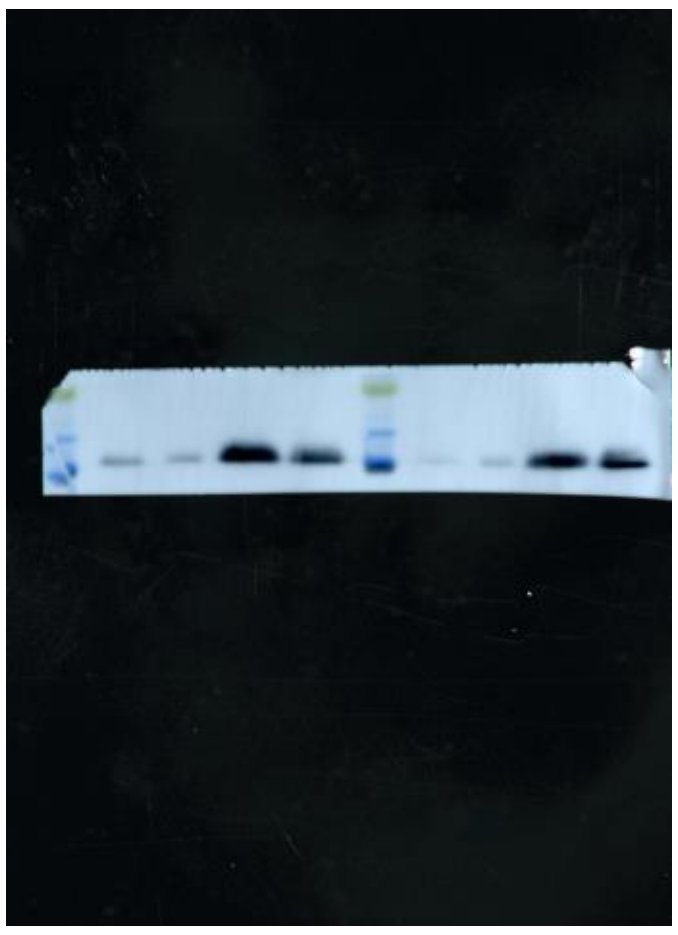

β-actin

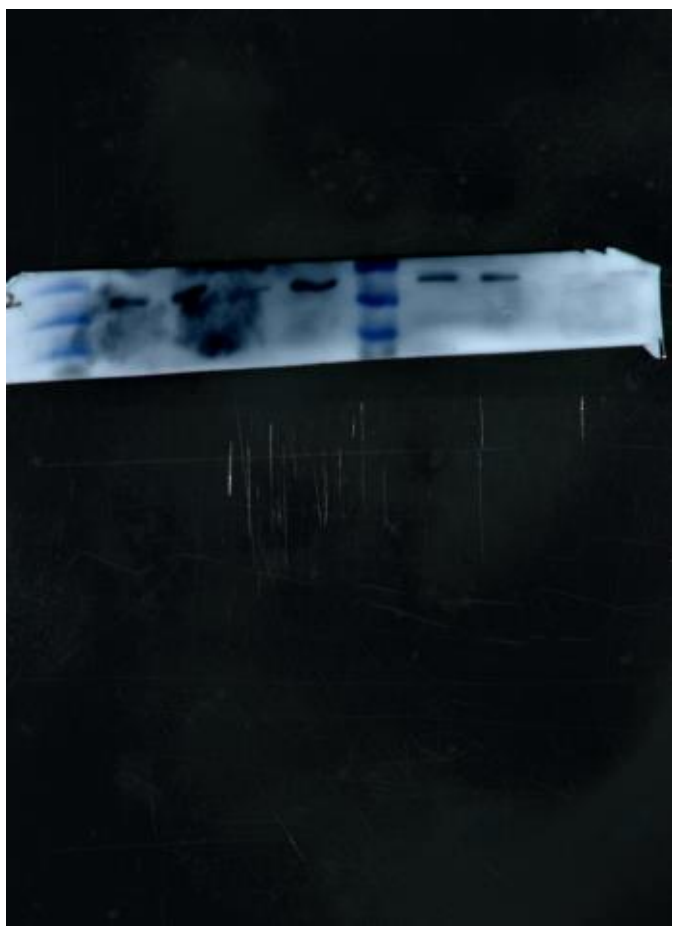

Fig.6A

H1703

pCaMKII（Thr286）

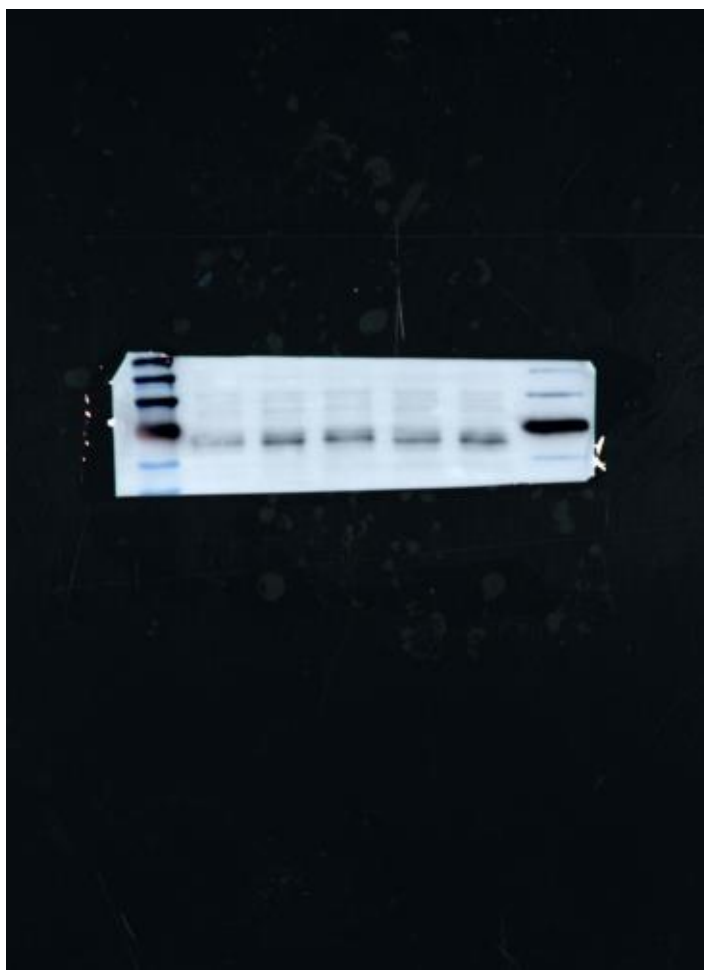

GAPDH

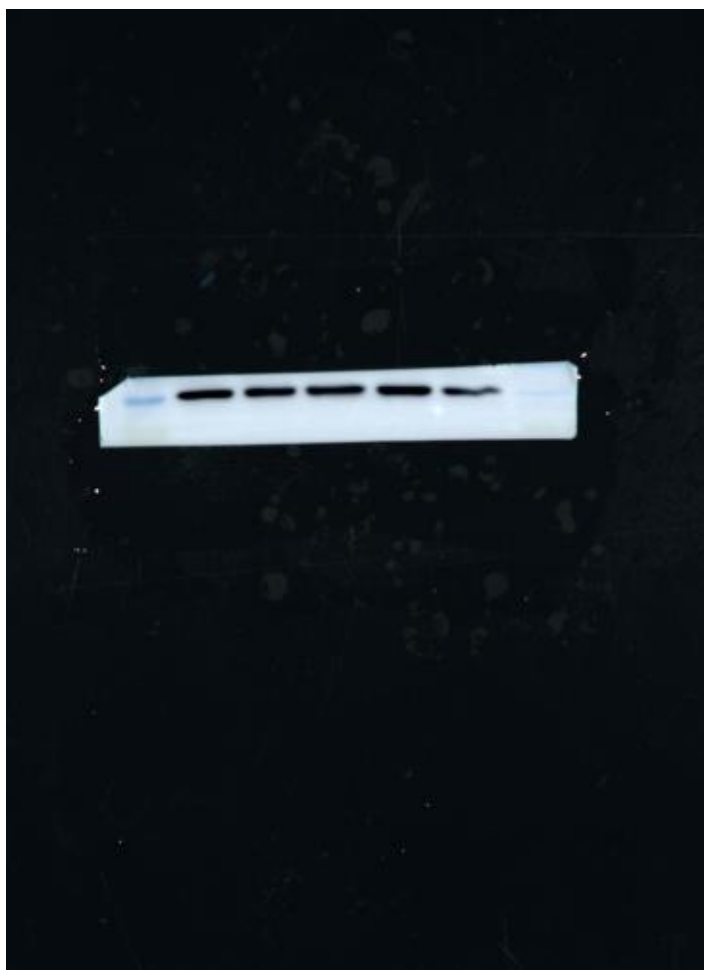

H520

pCaMKII（Thr286）& GAPDH

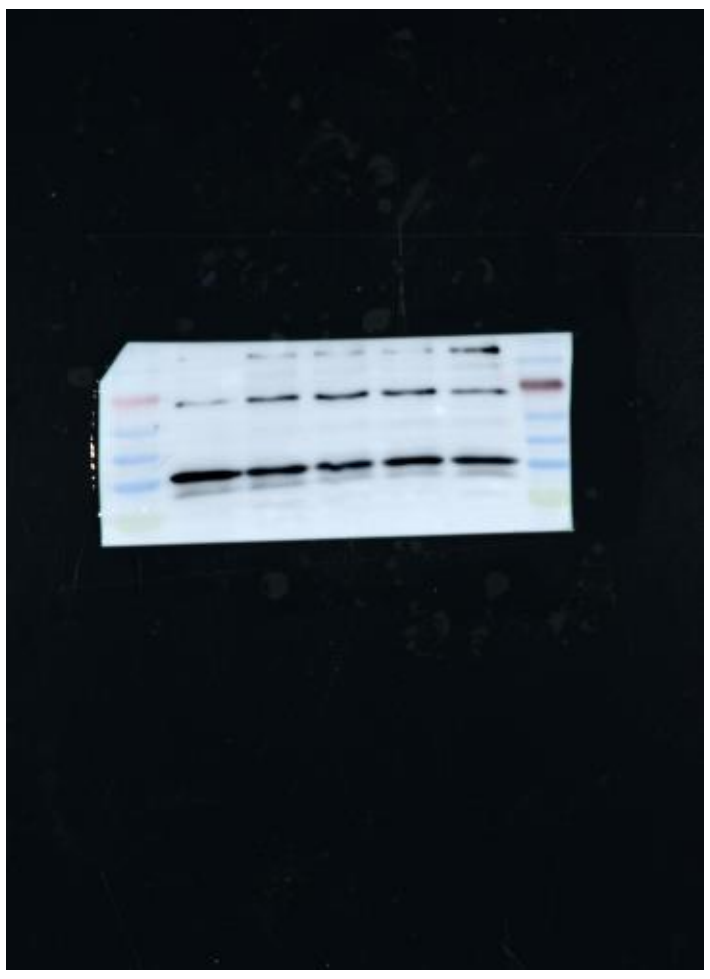

Fig.6B
H1703
pCaMKII（Thr286）

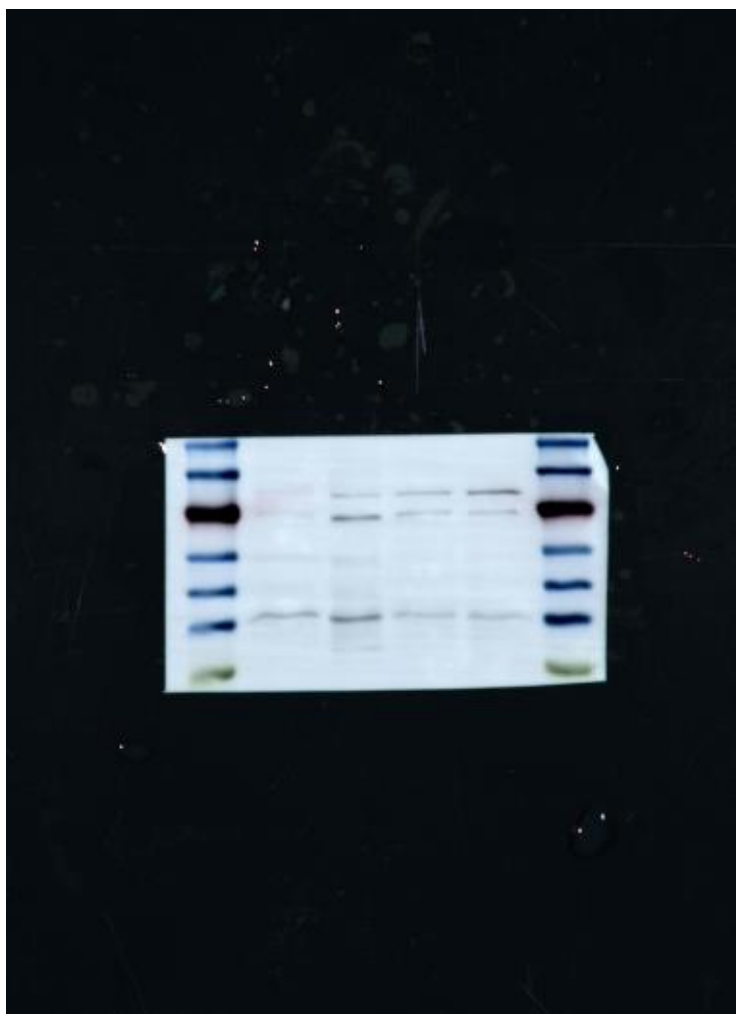

CaMKII

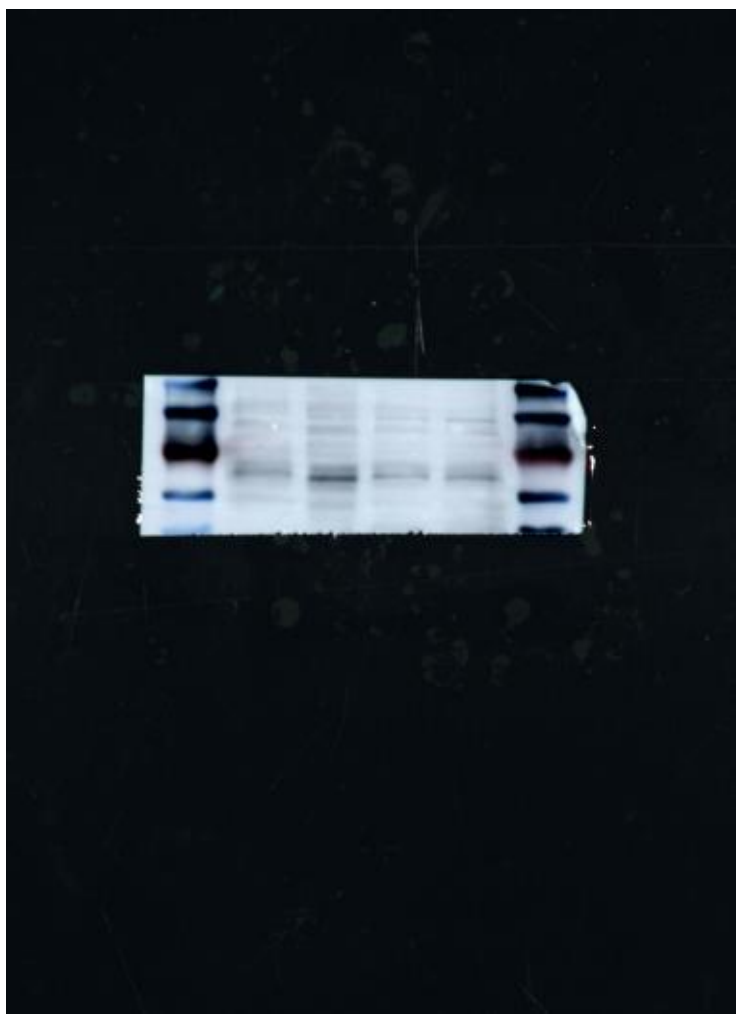

pDRP1（Ser616）& GAPDH

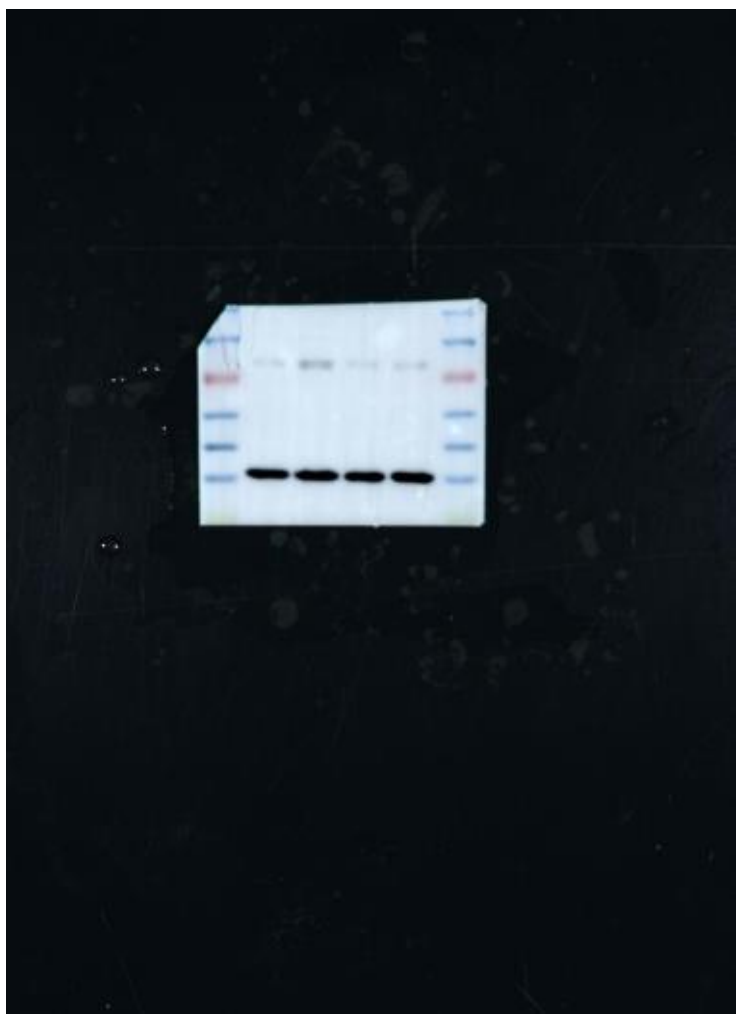

DRP1

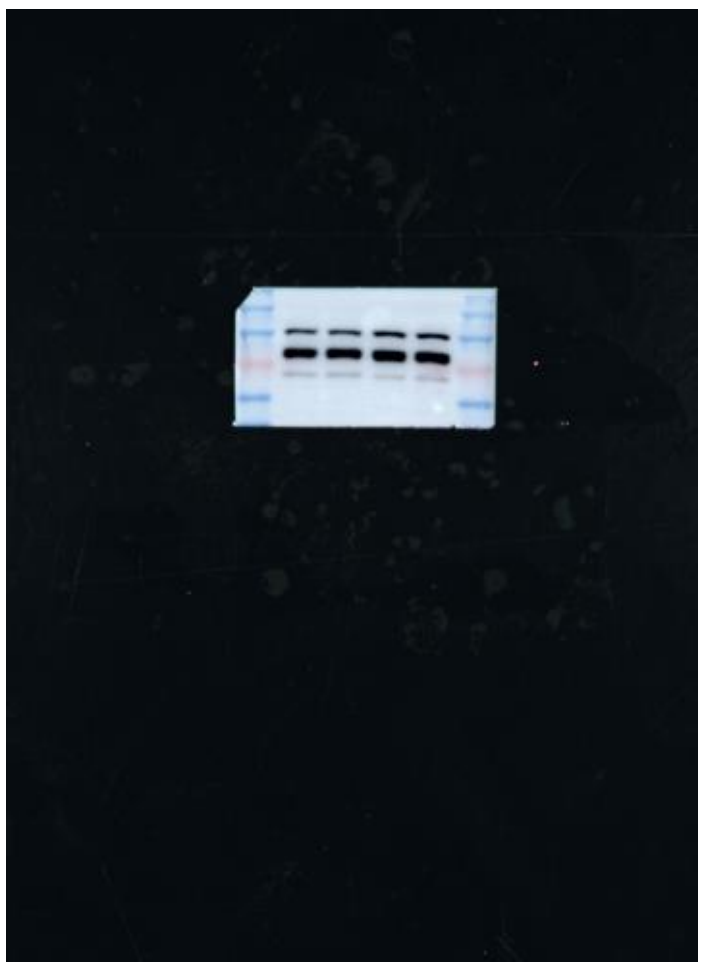

H520

pCaMKII（Thr286）

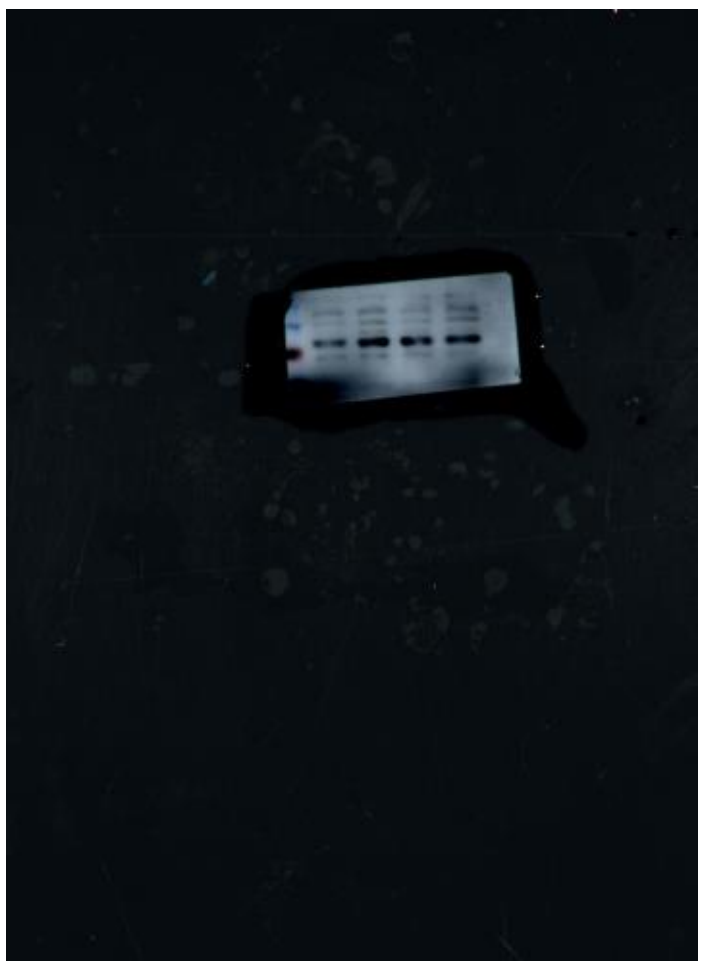

CaMKII

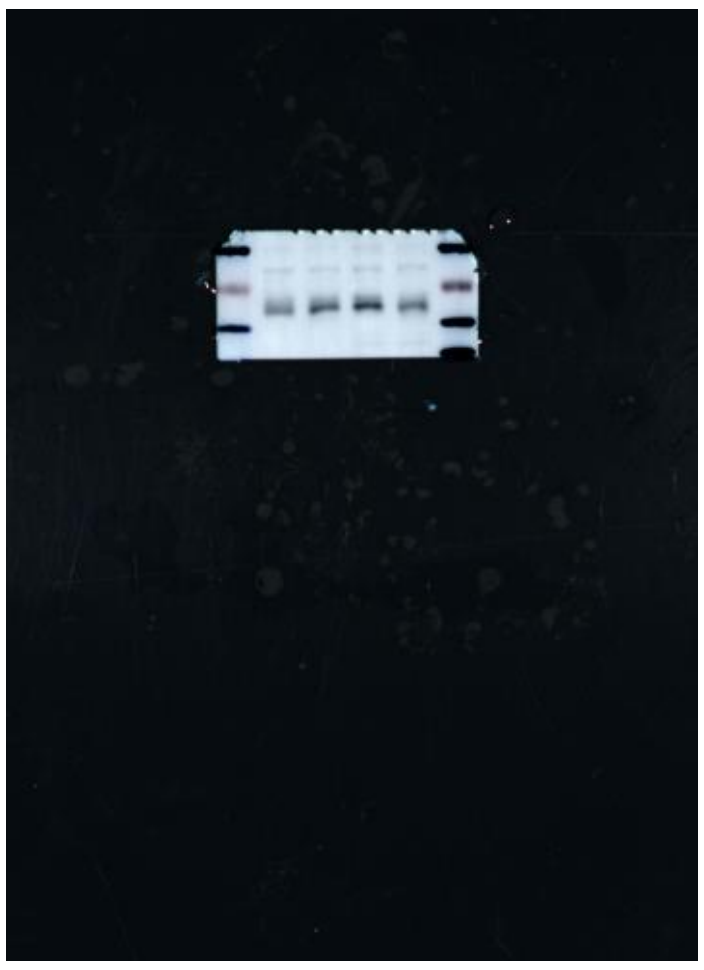

pDRP1（Ser616）

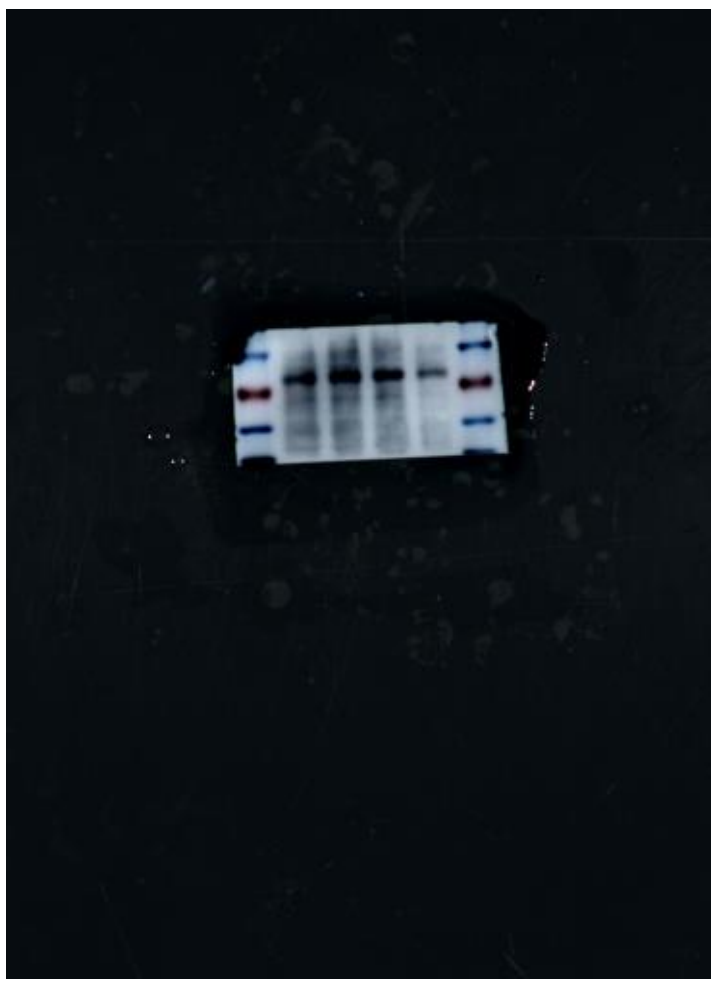

DRP1

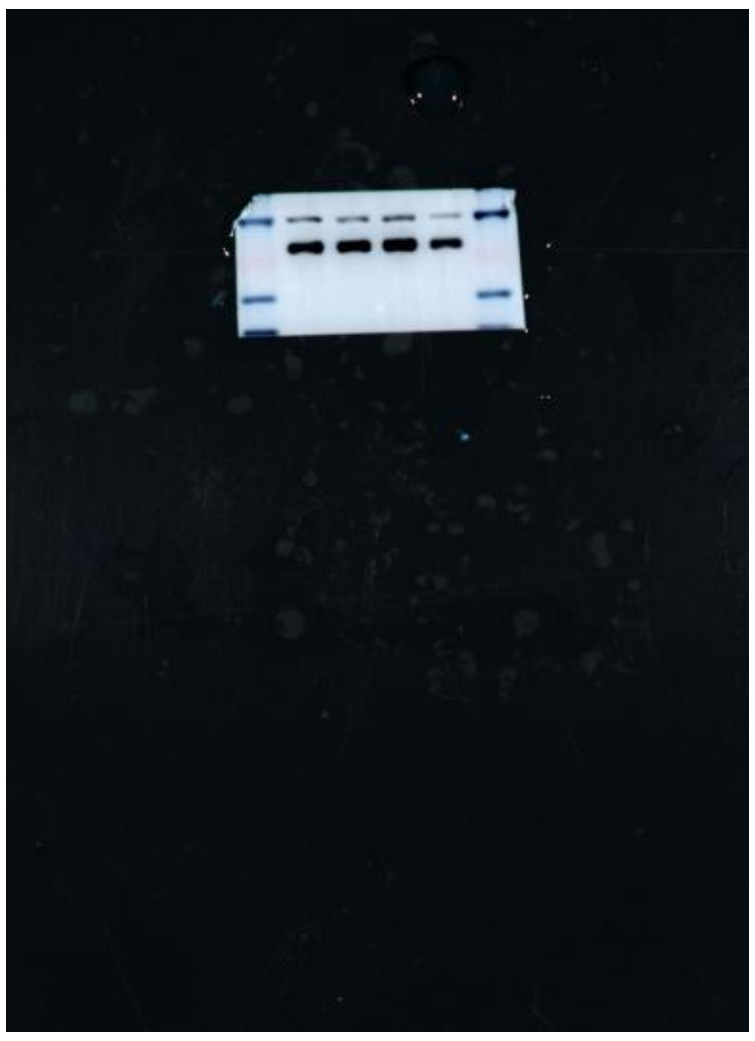

GAPDH

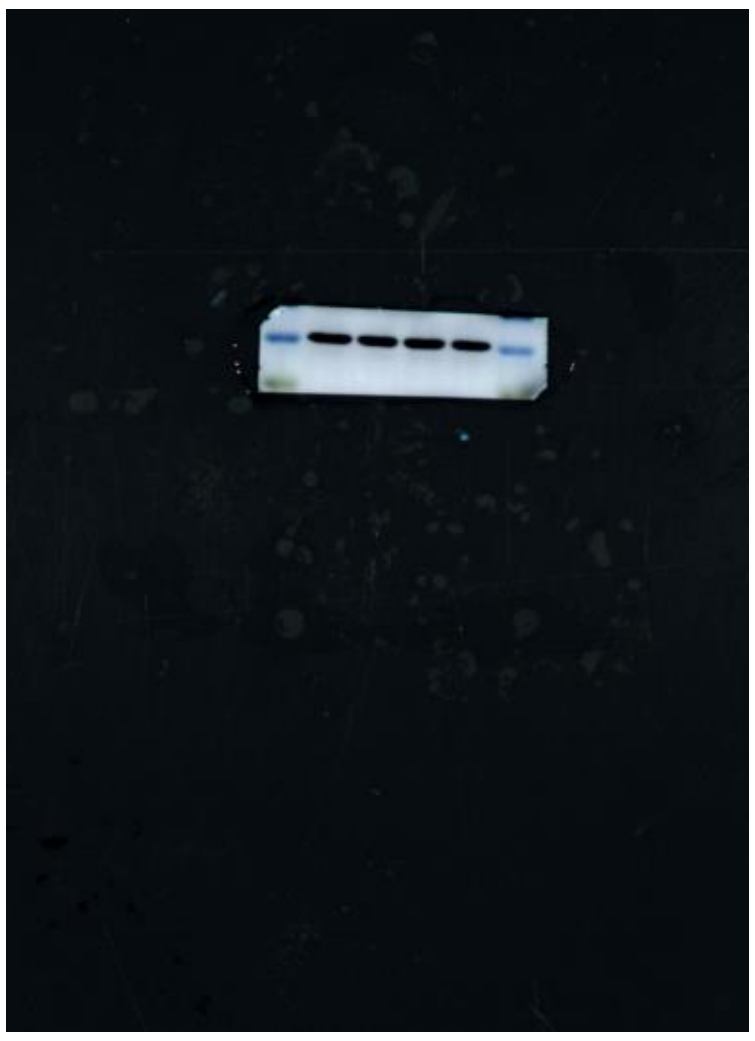

Fig.6F
H1703
GLUT1

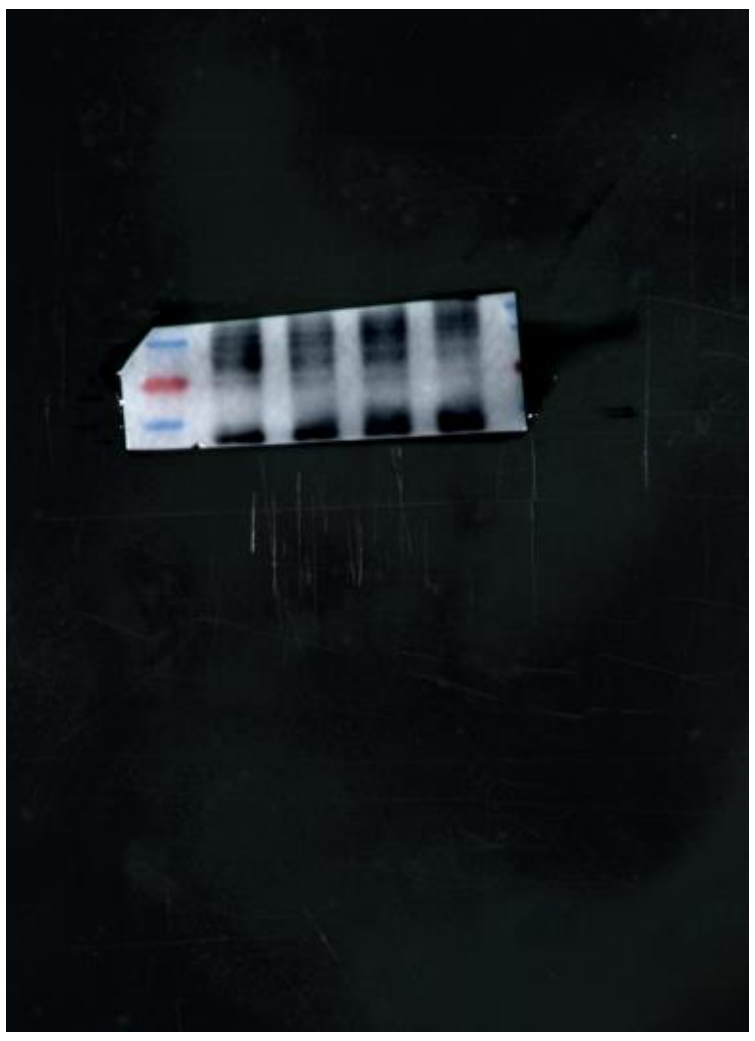

LDHA

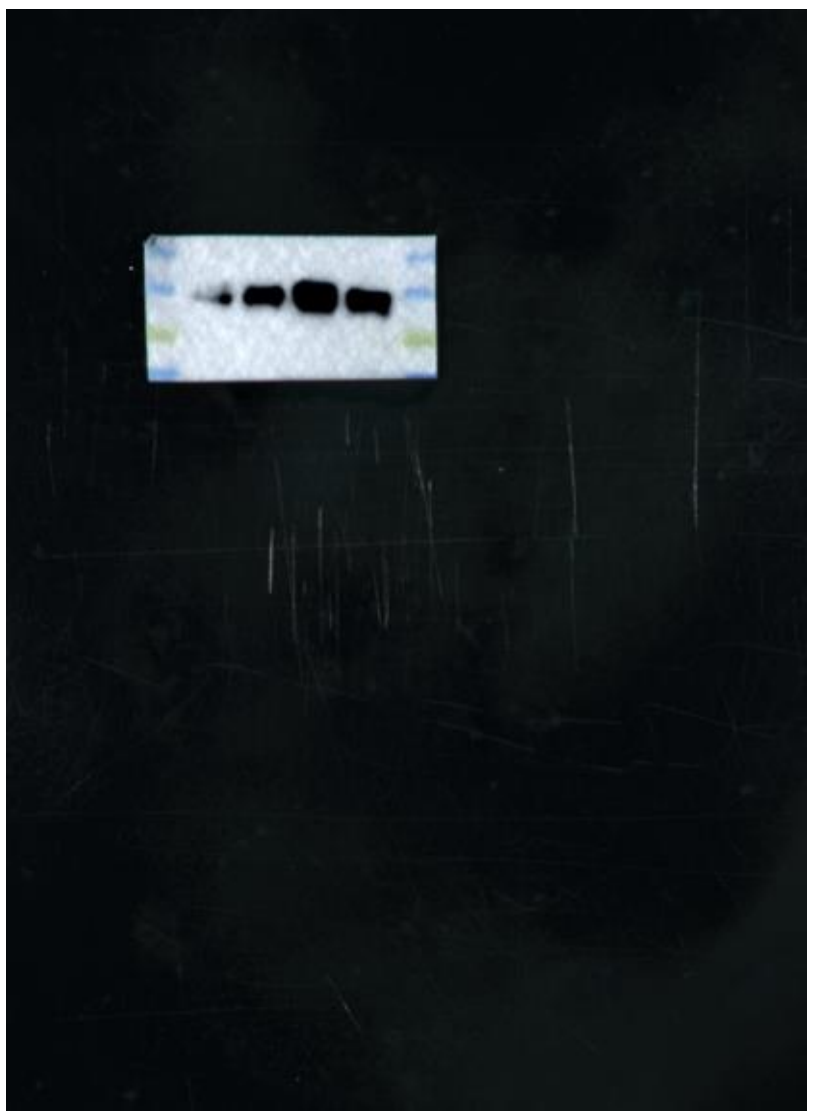

GAPDH

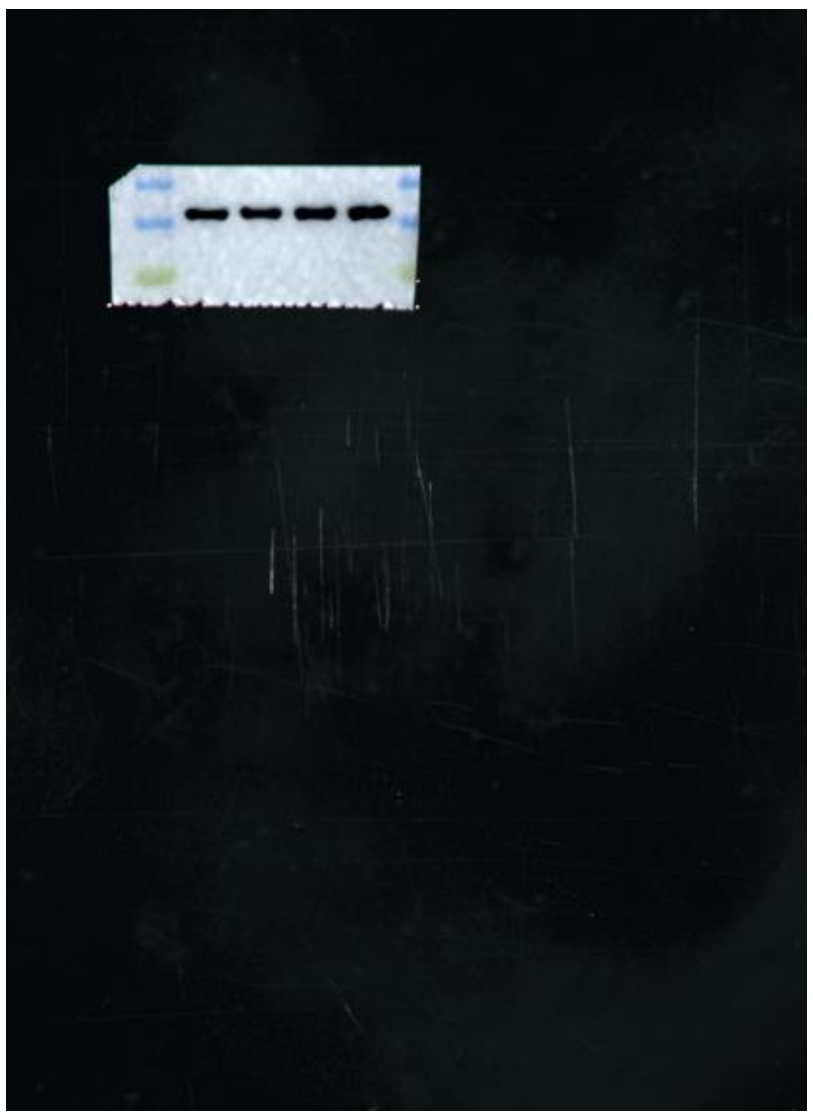

H520
GLUT1

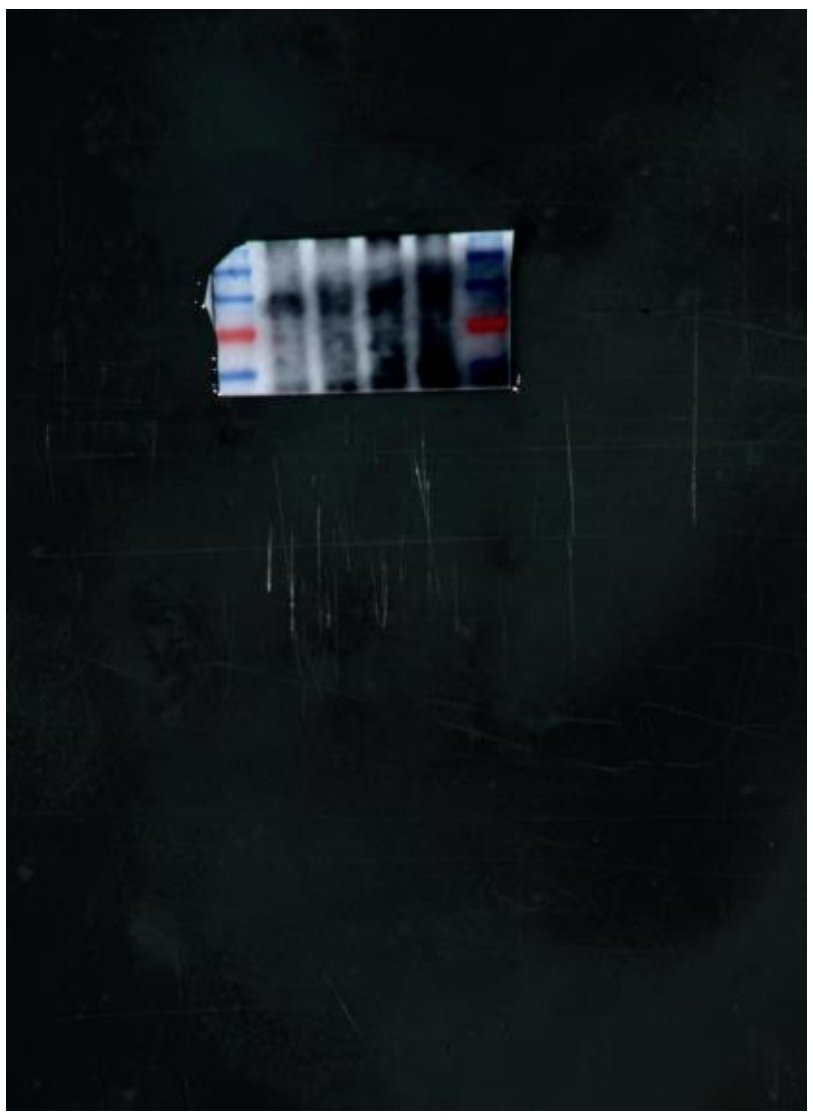

LDHA

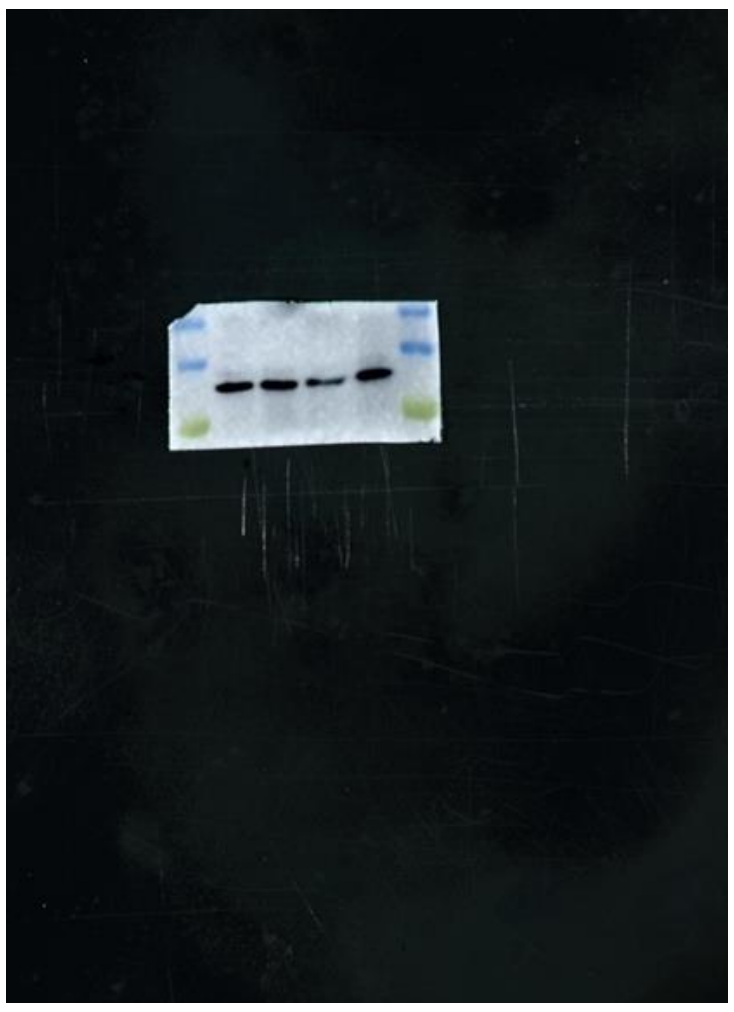

GAPDH

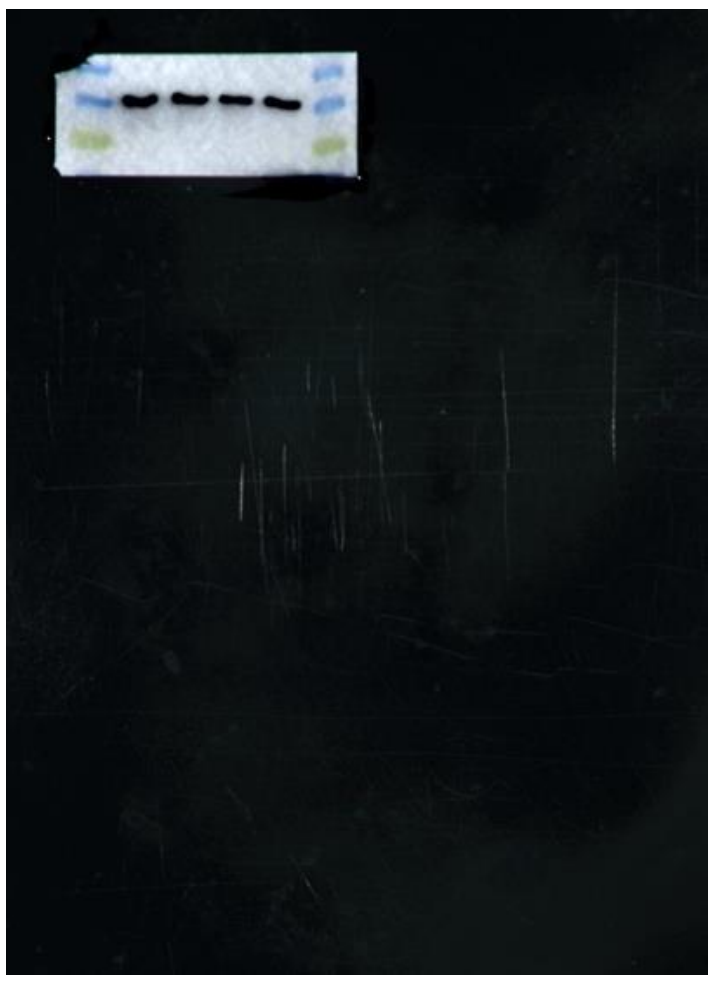

Supplement: Supplementary file 1 [file 41419_2026_9084_MOESM11_ESM.pdf]
