# Supplementary material for: Dual-faced metabolic reprogramming powers proliferation: FGF21 drives lung squamous carcinoma progression via CaMKII-DRP1-mediated mitochondrial fission
Source: Cell Death Dis. 2026 Jul 16;17(1):877. doi: 10.1038/s41419-026-09084-3 (PMC13655037; doi:10.1038/s41419-026-09084-3)
Supplement: Supplementary file 2 [file 41419_2026_9084_MOESM12_ESM.pdf]

Corresponding Author Name: Xiaojie Song
Manuscript Number: CDDIS-25-5015

SPRINGER NATURE

Reporting Summary

Springer Nature wishes to improve the reproducibility of the work that we publish. This checklist is used to ensure good reporting standards and to improve the reproducibility. Please respond completely to all questions relevant to your manuscript. For more information, please read the journal's Guide to Authors.

☑ **Check here to confirm that the following information is available in the Material & Methods section:**

- **The exact sample size (*n*)** for each experimental group/condition, given as a number, not a range
- **A description of the sample collection** allowing the reader to understand whether the samples represent technical or biological replicates (including how many animals, litters, culture, etc.)
- **A statement of how many times the experiment shown was replicated in the laboratory**
- **Definitions of statistical methods and measures**: For small sample sizes (n<5) descriptive statistics are not appropriate, instead plot individual data points
    - Very common tests, such as *t*-test, simple χ^2 tests, Wilcoxon and Mann-Whitney tests, can be unambiguously identified by name only, but more complex techniques should be described in the methods section
    - Are tests one-sided or two-sided?
    - Are there adjustments for multiple comparisons?
    - **Statistical test results**, e.g., ***P* values**
    - Definition of **'center values'** as **median or mean**;
    - Definition of **error bars as s.d. or s.e.m. or c.i.**

Please ensure that the answers to the following questions are reported in the manuscript itself. We encourage you to include a specific subsection in the methods section for statistics, reagents and animal models. Below, provide the page number or section and paragraph number.

Statistics and general methods

	**Reported in section/paragraph or page #**
1. How was the sample size chosen to ensure adequate power to detect a pre-specified effect size? (Give section/paragraph or page #)	Location:5.16. No formal power calculation was performed. Sample size for animal expriments(N=5 per group) was chosen based on common practice in xenografts studies. Location:5.16
For animal studies, include a statement about sample size estimate even if no statistical methods were used.	Location:5.16. (mice randomization,n=5/group); no explicit power analysis stated.
2. Describe inclusion/exclusion criteria if samples or animals were excluded from the analysis. Were the criteria pre-established? (Give section/paragraph or page #)	No samples or animals were excluced from analysis. Exclusioon criteria were not pre-established. Location: Not explicity reported; stated as “ none ” in this response.
3. If a method of randomization was used to determine how samples/animals were allocated to experimental groups and processed, describe it. (Give section/paragraph or page #)	Location:5.16. Mice were randomized into control and treatment groups after tumor reached 50-100mm^3. Randomization method is not detailed.
For animal studies, include a statement about randomization even if no randomization was used.	Location:5.16. Mice were randomized into control (PBS) and treatment (1ug/mouse rhFGF21) groups (n=5/groups)

4. If the investigator was blinded to the group allocation during the experiment and/or when assessing the outcome, state the extent of blinding. (Give section/paragraph or page #)	Location: 5.15. For mitochondrial morphology analysis, the investigator was blinded to treatment groups during image acquisition and quantification. No blinding started for other exprements.
For animal studies, include a statement about blinding even if no blinding was done.	Location: 5.15. All image files were coded and analyzed by an investigator blinded to the expremental
5. For every figure, are statistical tests justified as appropriate?	Yes.
Do the data meet the assumptions of the tests (e.g., normal distribution)?	Not formally tested.
Is there an estimate of variation within each group of data?	Yes (each figure legend states).
Is the variance similar between the groups that are being statistically compared? (Give section/paragraph or page #)	Not formally tested.

Reagents

	**Reported in section/paragraph or page #**
6. Report the source of antibodies (vendor and catalog number)	Provide in a table. 5.1, Table 1.(lists all antibodies with catalog numbers and vendoors)
7. Identify the source of cell lines and report if they were recently authenticated (e.g., by STR profiling) and tested for mycoplasma contamination	Location:5.4

Animal Models

	**Reported in section/paragraph or page #**
8. Report species, strain, sex and age of animals	Location:5.16
9. For experiments involving live vertebrates, include a statement of compliance with ethical regulations and identify the committee(s) approving the experiments.	Location:5.16 (first sentence) and Ethics Statement section.

10. We recommend consulting the ARRIVE guidelines (*PLoS Biol.* **8**(6), e1000412,2010) to ensure that other relevant aspects of animal studies are adequately reported.

Human subjects

Reported in section/paragraph or page #

11. Identify the committee(s) approving the study protocol.	Location:5.2 (human ethics), 5.16 (animal ethics),and Ethics Statement.
12. Include a statement confirming that informed consent was obtained from all subjects.	Location:5.2. Writtern informed consent was obtained from all study subjects.
13. For publication of patient photos, include a statement confirming that consent to publish was obtained.	No applicable - no patient photos are included in the manuscript.
14. Report the clinical trial registration number (at ClinicalTrials.gov or equivalent).	Not applicable - this is not a clinical trial.

15. For phase II and III randomized controlled trials, please refer to the CONSORT statement and submit the CONSORT checklist with your submission.

16. For tumor marker prognostic studies, we recommend that you follow the REMARK reporting guidelines.

Data deposition

Reported in section/paragraph or page #

17. Provide accession codes for deposited data. Data deposition in a public repository is mandatory for:<br>a. Protein, DNA and RNA sequences<br>b. Macromolecular structures<br>c. Crystallographic data for small molecules<br>d. Microarray data	Not applicable. All bioinformatics analyses were based on existing public databases(TCGA ,CCLE ,etc.), which are cited in the manuscript.

Deposition is strongly recommended for many other datasets for which structured public repositories exist; more details on our data policy are available in the Guide to Authors. We encourage the provision of other source data in supplementary information or in unstructured repositories such as Figshare and Dryad. We encourage publication of Data Descriptors (see Scientific Data) to maximize data reuse.

18. If computer code was used to generate results that are central to the paper's conclusions, include a statement in the Methods section under "**Code availability"** to indicate whether and how the code can be accessed. Include version information as necessary and any restrictions on availability.	Not applicable. All analyses were performed using standard commercial or open-source software (Graphpad Prism 9.0, R packages, Fiji, Seahorse Wave) as described in Section 5.18. No " Code availability " statement is included in the manuscript.
