# Supplementary material for: Dual-faced metabolic reprogramming powers proliferation: FGF21 drives lung squamous carcinoma progression via CaMKII-DRP1-mediated mitochondrial fission
Source: Cell Death Dis. 2026 Jul 16;17(1):877. doi: 10.1038/s41419-026-09084-3 (PMC13655037; doi:10.1038/s41419-026-09084-3)
Supplement: Supplementary file 3 [file 41419_2026_9084_MOESM1_ESM.docx]

Supplement Figure Legend

**Fig. S1**

(**A**) Pan-cancer analysis of FGF21 expression. TPM, transcripts per kilobase per million mapped reads; ACC, adrenocortical carcinoma; BLCA, bladder urothelial carcinoma; BRCA, breast invasive carcinoma; CESC, cervical squamous cell carcinoma and endocervical adenocarcinoma; CHOL, cholangiocarcinoma; COAD, colon adenocarcinoma; DLBC, lymphoid neoplasm diffuse large B-cell lymphoma; ESCA, esophageal carcinoma; GBM, glioblastoma multiforme; HNSC, head and neck squamous cell carcinoma; KICH, kidney chromophobe; KIRC, kidney renal clear cell carcinoma; KIRP, kidney renal papillary cell carcinoma; LAML, acute myeloid leukemia; LGG, brain lower grade glioma; LIHC, liver hepatocellular carcinoma; LUAD, lung adenocarcinoma; LUSC, lung squamous cell carcinoma; MESO, mesothelioma; OV, ovarian serous cystadenocarcinoma; PAAD, pancreatic adenocarcinoma; PCPG, pheochromocytoma and paraganglioma; PRAD, prostate adenocarcinoma; SARC, sarcoma; SKCM, skin cutaneous melanoma; STAD, stomach adenocarcinoma; TGCT, testicular germ cell tumors; THCA, thyroid carcinoma; THYM, thymoma; UCEC, uterine corpus endometrial carcinoma; UCS, uterine carcinoma; UVM, uveal melanoma. (**B-D**) OS and PFI analyses of FGF21 expression in LUSC. (**E**) Concentration of FGF21 in the serum of LUSC patients and healthy donors. (**F**) Correlation between FGF21 levels and mitochondrial function-related gene expression in LUSC patients.

**Fig. S2**

(**A**) Phosphorylated FGFR1 (pFGFR1) expression in LUSC cells following FGF21 treatment and/or AZD4547 treatment was analyzed by Western blotting.

**Fig. S3**

**(A)** Correlation between FGF21 levels and infiltrating tumor immune cell proportions across cancers. (**B**) Correlation between FGF21 levels and infiltrating tumor immune cell proportions in LUSC. (**C**) Correlations between FGF21 expression and T-cell gene markers in LUSC. (**D**) Correlations between FGF21 levels and macrophage gene markers in LUSC.

**Fig. S4**

(**A-B**) Dysregulated expression of DRP1 was observed in LUSC.

(**C**) Immunofluorescence colocalization of TOM20 and DRP1 in LUSC tissues.

(**D**) **Fis1 protein expression in H1703 and H520 cells treated with or without rhFGF21.** Representative Western blot images from **two independent experiments** are shown. GAPDH served as a loading control. Consistent trends of increased Fis1 expression were observed in both replicates; formal statistical analysis was not performed due to limited replicate number (n=2).

**Fig. S5**

(**A**) Western blot analysis of DRP1 protein levels in H1703 and H520 cells transfected with siRNA.

**Fig. S6 Baseline metabolic characterization of H1703 and H520 LUSC cell lines.**
**(A)** Basal oxygen consumption rate (OCR) to extracellular acidification rate (ECAR) ratio measured using a Seahorse metabolic analyzer. H1703 cells exhibited a significantly higher OCR/ECAR ratio compared to H520 cells, indicating a greater reliance on oxidative phosphorylation at baseline. Data are presented as mean ± SD from three independent experiments. **p < 0.01.
**(B)** Comparative analysis of key glycolytic gene expression (LDHA, PKM2, HK2, PFKM) between H1703 and H520 cells based on Cancer Cell Line Encyclopedia (CCLE) RNA-seq data. H520 cells show higher expression of glycolytic enzymes relative to H1703 cells. Data are presented as log2(TPM+1).

**Table S1** Concentration of FGF21 in the serum of LUSC patients and healthy donors.

**Table S2** DEGs associated with FGF21 in LUSC tumor samples.

**Table S3** H-scores of DRP1 in LUSC tumor and adjacent non-tumor tissues.
